# Electrofluorochromic Switching of Heat-Induced Cross-Linkable Multi-Styryl-Terminated Triphenylamine and Tetraphenylethylene Derivatives

**DOI:** 10.3390/molecules29102340

**Published:** 2024-05-16

**Authors:** Kang Le Osmund Chin, Pin Jin Ong, Qiang Zhu, Jianwei Xu, Ming Hui Chua

**Affiliations:** 1 Institute of Sustainability for Chemicals, Energy and Environment (ISCE2), Agency for Science, Technology and Research (A*STAR), 1 Pesek Road, Jurong Island, Singapore 627833, Singapore; osmund_chin@isce2.a-star.edu.sg (K.L.O.C.); zhuq@imre.a-star.edu.sg (Q.Z.); 2 Institute of Materials Research and Engineering (IMRE), Agency for Science, Technology and Research (A*STAR), 2 Fusionopolis Way, Innovis, #08-03, Singapore 138634, Singapore; ong_pin_jin@imre.a-star.edu.sg; 3 Department of Chemistry, National University of Singapore (NUS), 3 Science Drive 3, Singapore 117543, Singapore

**Keywords:** electrofluorochromic, electrochromic, aggregation-induced emission, cross-linking monomers, styryl functionalization

## Abstract

High-performance electrochromic (EC) and electrofluorochromic (EFC) materials have garnered considerable interest due to their diverse applications in smart windows, optoelectronics, optical displays, military camouflage, etc. While many different EC and EFC polymers have been reported, their preparation often requires multiple steps, and their polymer molecular weights are subjected to batch variation. In this work, we prepared two triphenylamine (TPA)-based and two tetraphenylethylene (TPE)-based derivatives functionalized with terminal styryl groups via direct Suzuki coupling with (4-vinylphenyl)boronic acid and vinylboronic acid pinacol ester. The two novel TPE derivatives exhibited green–yellow aggregation-induced emission (AIE). The EC and EFC properties of pre- and post-thermally treated derivatives spin-coated onto ITO–glass substrates were studied. While all four derivatives showed modest absorption changes with applied voltages up to +2.4 V, retaining a high degree of optical transparency, they exhibited obvious EFC properties with the quenching of blue to yellow fluorescence with I_OFF/ON_ contrast ratios of up to 7.0. The findings therefore demonstrate an elegant approach to preparing optically transparent, heat-induced, cross-linkable styryl-functionalized EFC systems.

## 1. Introduction

Materials exhibiting electrochromic (EC) and electrofluorochromic (EFC) properties can have their color and fluorescence, respectively, modulated by applying an external electrical bias. Such materials are highly sought after for applications in smart windows, antiglare mirrors, optoelectronics, optical displays, military camouflage, etc. [1]. Evolving from the very first report of electrochromism found in tungsten oxide, made by Deb in 1969 [2], there have since been myriad EC materials reported, from metal oxides [3,4,5] to metal complexes [6,7,8,9], small molecules [10,11,12,13], and organic polymers [14,15,16,17,18], which enable the switching of a wide range of colors across the ultraviolet (UV) to near-infrared (NIR) spectrum. This reflects the immense interest in this field of research fueled by the high demand for such materials. Particularly for organic polymers, it has been well illustrated that color tuning can be achieved by structural modulation, especially in conjugated polymers, using different donor and acceptor groups as building blocks [19,20,21]. Electrofluorochromism, on the other hand, has garnered increasing attention recently due to the applicability of EFC materials in optoelectronics, smart optical displays, and even in chemical and biological sensing [22,23,24,25,26]. Likewise, EFC switching of fluorescence wavelengths across the entire visible region has become possible with the reports of numerous electroactive luminogens in the forms of metal complexes, small molecules, and conjugated and non-conjugated polymers [27,28,29,30].

Like other light-emitting optoelectronic devices and applications, the performance of EFC devices will benefit tremendously from aggregation-induced emission (AIE) properties inherent in EFC materials. Unlike conventional luminogens, which exhibit aggregation-caused quenching (ACQ) properties, an AIE-active compound in general is non-emissive or emits weakly in dilute solutions, but emissions can be turned on or dramatically enhanced in the aggregated or solid state, attributed to the restriction of intramolecular motion (RIM), including rotation and vibration. Such compounds have been widely studied due to their vast usefulness in a wide range of applications in the areas of optoelectronics, sensing, and biomedicine [31,32,33,34,35,36]. In this regard, there have been several recent reports of AIE-active EFC materials, both small molecules and polymers, many of which incorporate triphenylamine (TPA) and tetraphenylethylene (TPE) as electroactive and AIE-active building blocks, respectively, in rational structural designs [25,37,38,39,40,41,42,43]. With growing attention devoted to both fields, it is envisaged that there will be increasing research activity and interest in developing novel AIE-active EFC materials, aiming to enhance EFC switching performance and control emission wavelengths.

Organic optoelectronic materials possess several advantages over their inorganic counterparts, and these include light weight, flexibility, solution processibility, potentially lower toxicity, lower cost, and abundance of raw materials [44,45,46]. However, their preparation often involves multiple-step synthesis, and in the case of polymers, molecular weights may vary between potentially influencing material performances. Issues of poor solubility of highly cross-linked or multidimensional macromolecules may also complicate solution processibility. To address the two latter issues, in situ polymerization approaches, such as electro-polymerization of monomers onto device substrates, UV curing, and heat treatment of monomer-coated substrates, have been commonly adopted.

The styryl group is known to easily polymerize under UV irradiation or heating into polystyrene, serving as a viable approach to prepare cross-linkable polymer networks. Joseph et al. reported a bis(diphenyl-amino)TPE derivative decorated with two 4-vinylbenzyloxy groups [47]. Thin films spin-coated with this derivative can form a cross-linked network upon heating at 215 °C for 30 min. An EC device (ECD) fabricated with heat-treated films exhibited a transmissive-to-black color change and quenching of yellowish-green fluorescence with the application of positive voltages up to +2.8 V. A similar approach to developing thermally cross-linked polymers for highly transparent EC materials with terminal styryl functional groups was also recently reported by Zhang’s group [48]. Inspired by the above work, we wish to explore alternative approaches that may be equally or more convenient and straightforward to develop styryl-functionalized cross-linkable derivatives for EC and EFC applications, with the inclusion of AIE properties to enhance performance for the latter.

Here, four novel styryl tris-functionalized TPA (**M1** and **M2**) and octa-functionalized tetrakis(diphenylamine)-TPE (**M3** and **M4**) derivatives were synthesized via direct Suzuki coupling with vinyl and styryl boronic acids/esters. Spin-coated thin films of **M1** to **M4** underwent cross-linking via thermal treatment. While all four derivatives, whether heated or not, exhibited modest absorption changes with increasing positive voltage applied, obvious quenching of blue to yellow fluorescence with I_OFF/ON_ contrast ratios of up to 7.0 was observed. The structure–property relationship between AIE, EC, and EFC properties of the four derivatives **M1** to **M4** and in particular the effects of heat-induced cross-linking on their EC and EFC properties were investigated, showing clear optical (both absorption and emission) changes of cross-linkable **M1** to **M4** upon heat treatment. Therefore, this work demonstrates the potential in the synthesis and application of heat-induced cross-linkable compounds to tune and subsequently achieve better EC and EFC properties.

## 2. Results and Discussion

**Scheme 1:**
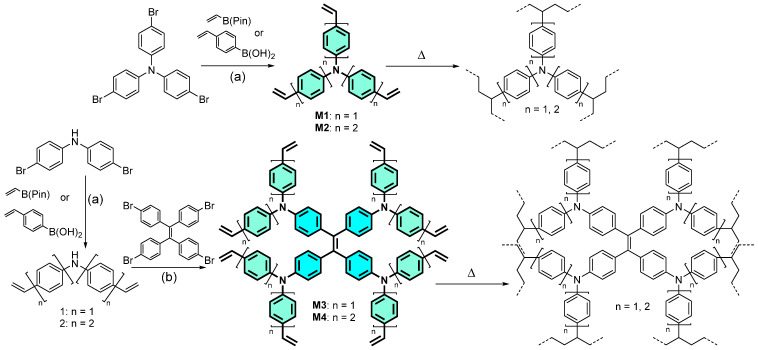
Synthesis of styryl-terminated TPA and TPE derivatives **M1**–**M4** and proposed subsequent cross-linking polymerization upon undergoing thermal treatment. Reagents and conditions: (a) Pd(PPh_3_)_4_, K_2_CO_3_, toluene, ethanol, water, 95 °C for 18 h; (b) Pd(OAc)_2_, KO(t-Bu), P(t-Bu)_3_, toluene, reflux for 18 h.

Four styryl-terminated TPA and TPE derivatives, **M1**–**M4**, were prepared starting from readily available precursors via straightforward C-C and C-N coupling reactions (Scheme 1). TPA is a common electroactive moiety, serving as a building block for EC and EFC systems. First, TPA was functionalized with multiple vinyl and styryl groups, simply by Suzuki coupling with vinylboronic acid pinacol ester and 4-vinylphenylboronic acid to afford cross-linkable derivatives **M1** and **M2**, respectively, in good yields, both of which contain three terminal styryl groups.

On the other hand, TPE is a classic AIE luminogen which serves as an important scaffold in the designs of many different AIE-active systems and functional materials. We believe that introducing AIE properties into EFC material could facilitate the enhancement of EFC switching properties, particularly the switch-off/switch-on contrast ratios (I_OFF_/_ON_). Envisaging an AIE-active EFC system, highly branched **M3** and **M4** containing eight terminal styryl groups were designed to incorporate both AIE and electroactive elements of TPE and TPA, respectively. The synthesis of **M3** and **M4** involved a facile two-step process via Suzuki coupling reactions with vinylboronic acid pinacol ester and 4-vinylphenylboronic acid to bis(4-bromophenyl)amine to afford intermediates bis(4-vinylphenyl)amine (**1**) and bis(4′-vinyl-[1,1′-biphenyl]-4-yl)amine (**2**), respectively. Then, intermediates **1** and **2** were coupled onto the TPE moiety via Buchwald–Hartwig amination with tetrabromo-TPE.

Subsequent heating of derivatives **M1**–**M4** may lead to cross-linking via polymerization of the terminal styryl groups. This is evident from FTIR analysis, which shows a decrease in styryl alkene C-H peaks at 1598–1597 cm^−1^ and 1505–1493 cm^−1^, out-of-plane alkene bend at 841–821 cm^−1^, and alkene C-H stretch at 3100–3000 cm^−1^ (Figure S1, ESI).

TPA derivatives **M1** and **M2** both appear as off-white solids, whereas diphenylamine-TPE derivatives **M3** and **M4** appear as bright greenish-yellow and yellow solids, respectively. Table 1 summarizes the optical and electrochemical properties of **M1** to **M4**. Both **M1** and **M2** solutions absorb in the longwave ultraviolet (UV-A) region with a single absorption band of exhibiting maximum absorption (λ_abs_) at 350 and 359 nm, respectively. The slight redshift in the absorption profile of **M2** compared to that of **M1** is attributable to extension of π-conjugation arising from one additional phenyl ring between the terminal vinyl group and the TPA core. This also leads **M2** to have a slightly longer absorption onset wavelength (λ_onset_) of 406 nm (vs. 385 nm) translating to a narrower optical bandgap (E_g_^opt.^) of 3.05 eV.

With four diphenylamine groups decorated over TPE, **M3** and **M4** are expected to possess more extensive π-conjugation networks compared to **M1** and **M2**, which translates to red-shifting of absorption profiles. Even though there are only modest redshifts in λ_abs_ for **M3** and **M4**, their solution absorption spectra clearly extend beyond UV-A into the visible blue region with λ_onset_ of 457 and 462 nm, respectively, and with the former also displaying a shoulder peak at ca. 425 nm (Figure S2, ESI). This contributes to even narrower E_g_^opt.^ of 2.71 and 2.68 eV for **M3** and **M4**, respectively. Likewise, with additional phenyl rings, **M4** expectedly exhibit redshifted λ_abs_ and λ_onset_ compared to **M3**. Furthermore, **M3** and **M4** possess a significantly larger molar absorptivity (ε) compared to **M1** and **M2**, which similarly can be attributed to their more extensive π-conjugation. With the exception of **M1**, all derivatives showed a slight redshift in thin-film absorption compared to solution absorption spectra, which can be attributed to the effect of molecular packing. Cyclic voltammetry (CV) studies show that the HOMO energies of **M1**–**M4** are rather consistent: in the range of −4.92 to −5.03 eV (Figure S3, ESI). Deriving their respective LUMO energies from E_g_^opt.^, a trend of decreasing LUMO from −1.72 to −2.29 eV was observed going from **M1** to **M4**.

The fluorescence properties of **M1**–**M4** were examined. When excited at their respective λ_abs_, solutions of **M1** and **M2** emit deep blue fluorescence with emission maxima wavelengths (λ_FL_) of 405 and 435 nm, respectively. Similarly, with a greater extent of π-conjugation arising from additional phenyl rings, the emission profile of **M2** appears more redshifted than that of **M1**. Meanwhile, **M3** and **M4** solutions exhibit weak-yellowish fluorescence, with the former revealing a single emission band with λ_FL_ at 533 nm, and the latter two emission bands with λ_FL_ at 430 and 533 nm, respectively.

**Figure 1:**
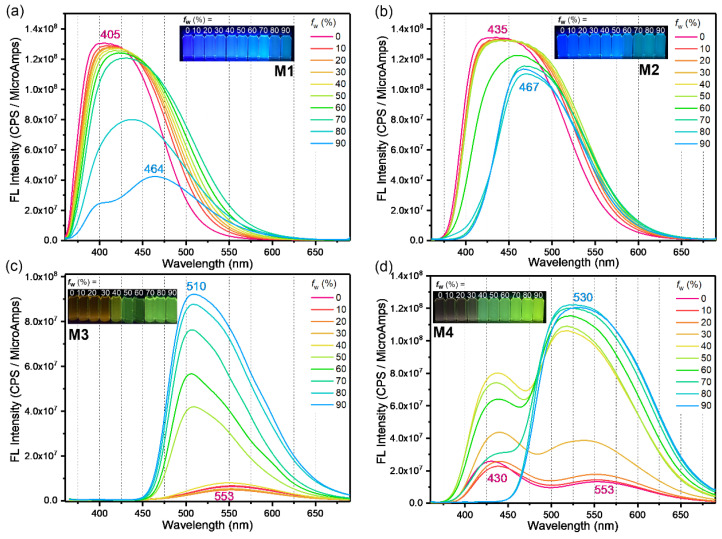
Fluorescence spectra of 0.1 mM of (**a**) **M1**, (**b**) **M2**, (**c**) **M3**, and (**d**) **M4** solutions in THF–water binary solvent mixtures of different proportions (*f*_w_ denotes water fractions). Inserts are photos of the respective solutions taken under UV irradiation of 365 nm.

The effects of aggregation on the fluorescence properties of **M1**–**M4** were evaluated by recording their fluorescence spectra under the same concentration in binary solvent mixtures with different proportions of THF (good solvent) and water (anti-solvent), in which increasing water fractions (*f*_w_) will cause the molecules to aggregate. Although TPA and its derivatives **M1** and **M2** possess fan-like structures with phenyl rings freely rotatable about the central nitrogen atom, their trigonal arrangements are insufficient to confer AIE properties, probably because adjacent phenyl rings are too widely spaced apart to hinder π–π stacking upon aggregation. As such, **M1** exhibits moderate ACQ properties in which fluorescence spectra redshift and quench to approximately a third of the initial intensity as *f*_w_ increases from 0 to 90% (Figure 1a). Meanwhile, **M2** exhibits mild ACQ behavior in which the increase in *f*_w_ from 0 to 90% only leads to a slight quenching of fluorescence, with the fluorescence quantum yield (**Φ**_FL_) decreasing from 13.6% to 10.8%, possibly due to the additional phenyl rings imposing slightly more RIM and hindering π–π stacking upon molecular aggregation. This is accompanied by slight red-shifting of λ_FL_ from 435 to 467 nm, in which aggregated **M2** solutions appear to emit weaker bluish-green fluorescence (Figure 1b).

The presence of TPE in the molecular scaffold and highly congested structures contributes to the obvious AIE character of **M3** and **M4**. Both derivatives reveal a turning on of greenish-yellow fluorescence as *f*_w_ increases from 0 to 90% (Figure 1c,d). The fluorescence intensity of **M3** increases ca. 140 times from **Φ**_FL_ of 0.08% to 11.2% with a blueshift in λ_FL_ from 553 to 510 nm. **Φ**_FL_ of **M4** increases ca. 95-fold from 0.15% to 14.2%. Interestingly, the emission bands at both 430 and 553 nm initially increase in intensity as *f*_w_ increases from 0 to 40%, but subsequently, the former starts to collapse while the latter continues to enhance and blue-shift as *f*_w_ increases to 90%. Aggregated solution of **M4** presents as a single broad emission band with λ_FL_ at 530 nm.

Styryl functionalized derivatives may undergo photopolymerization under UV curing in the presence of a photoinitiator, or simply by heating. We opt for the latter to avoid possible complications towards device performance due to the additional presence of trace photoinitiator, as well as the relative safety and convenience of the latter approach. **M1** and **M4** are highly soluble in common organic solvents, rendering them highly solution processible. They were spin-coated onto ITO–glass substrates for subsequent heat treatment and device fabrication. Spin-coated substrates were heated at 215 °C under a N_2_ atmosphere to promote cross-linking.

**Figure 2:**
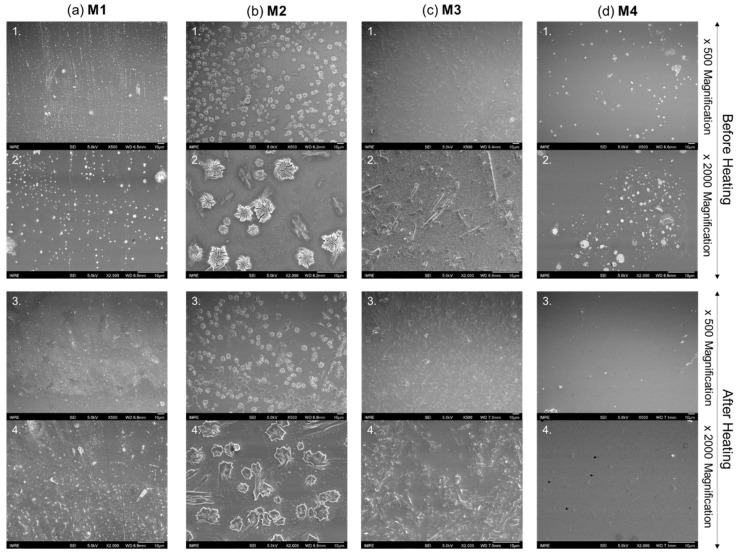
SEM images of (**a**) **M1**, (**b**) **M2**, (**c**) **M3**, and (**d**) **M4** thin films before and after heating.

Figure 2 shows the SEM images of pre- and post-heated **M1**–**M4** thin films. Pre-heated thin films of **M1**, **M2** and **M4** show a rather granular morphology, with the grain distributions in **M1** and **M2** being more homogeneous. The grain particles of **M2** are also larger and more plate-like, whereas those of **M1** and **M4** appear more globular. Pre-heated thin film of **M3** appears very homogeneous with a slight fibrous texture. Thirty minutes of heat treatment led to observable morphology changes in all four derivatives, likely due to the cross-linking of the terminal styryl groups leading to changes in molecular packing structure. The film morphologies of **M1** and **M4** became less grainy, particularly for the latter, in which most of the grain particles disappeared into a smoother and more homogeneous morphology. Meanwhile, the opaque platy grain particles of **M2** became more translucent and developed smoother edges upon heating, whereas the surface morphology of **M3** appears more fibrous in texture.

We first studied the effects of heating and cross-linking on the EC properties of **M1**–**M4**. Heating of spin-coated thin films results in a slight blueshift of absorption due to cross-linking of terminal styryl groups, causing a slight decrease in the extent of π-conjugation when the sp^2^ vinyl groups convert into sp^3^ alkane groups (Figure S4, ESI). Due to their minimal absorption in the visible region, thin films and hence fabricated ECDs of pre- and post-heated **M1**–**M4** appear almost optically transparent, with pre-heated **M3** and **M4** films having a slight yellow tint, which became even less obvious upon heating.

**Figure 3:**
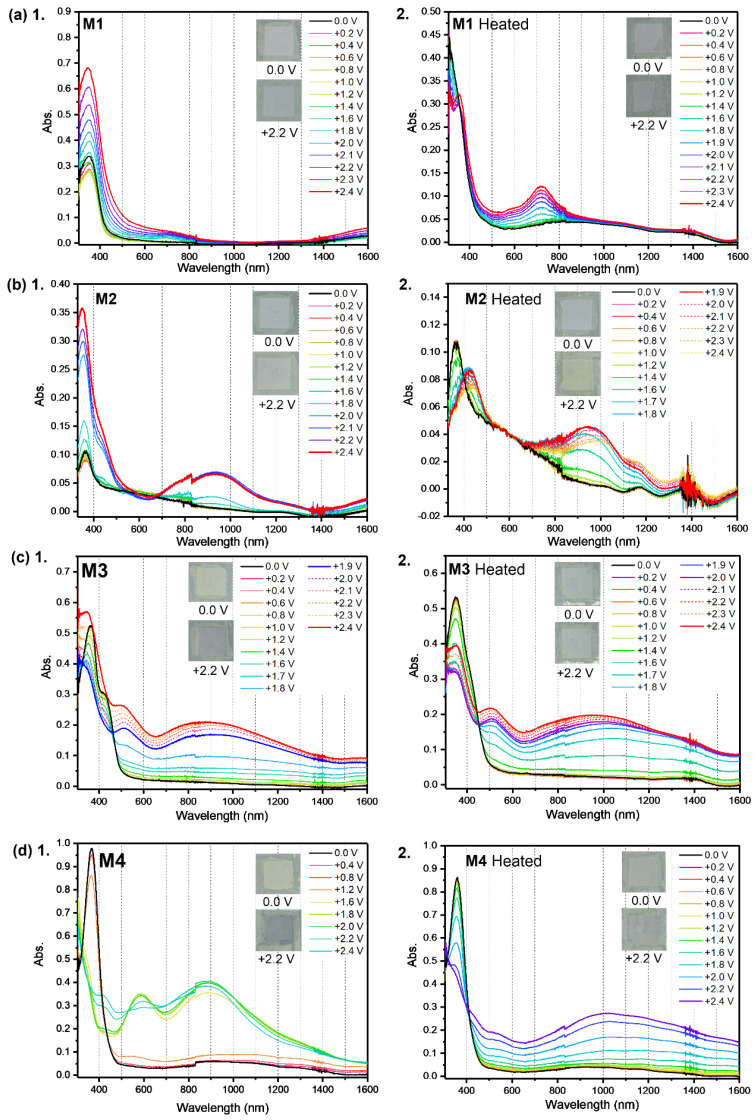
EC spectroelectrochemistry of ECDs fabricated from (**a**) **M1**, (**b**) **M2**, (**c**) **M3**, and (**d**) **M4**. Insert: photos showing colors of the respective ECDs at 0.0 and +2.2 V.

Spectroelectrochemistry studies were performed on fabricated ECDs by measuring absorbance between 300 and 1600 nm while applied voltages were increased from 0.0 to +2.4 V. Meanwhile, there were no changes in absorbance with increasing negative voltages applied. Increasing applied voltage is believed to cause electro-oxidation of the electroactive TPA groups, which can then form radical mono-cations and di-cations. Modest changes in the absorbance in the visible and NIR region were observed for pre-heated **M1**, as increasing applied voltages led to continuous increase in intensity of a ca. 360 nm absorption peak, as well as the emergence of a very low intensity band at ca. 700 nm at +1.8 V. In comparison, heated **M1**, however, showed the emergence of a more obvious absorption band at ca. 720 nm from applied voltages +1.6 V onwards (Figure 3a). Pre-heated **M2** showed a similar increase in UV-A absorbance at ca. 350 nm with increasing applied voltages accompanied by the emergence of a more obvious absorption band at ca. 950 nm at the onset of +1.6 V and intensity peaking at +2.1 V. The emergence of a new 950 nm absorption band was also observed for heated **M2** but appeared at an earlier-onset applied voltage of +1.2 V and peaked in intensity at +1.9 V. Subsequent increases in applied voltages led to the collapse and red-shifting of this absorption band (Figure 3b). In addition, oxidized ECDs fabricated from heated-**M2** revealed a slight yellow tint in coloration, while the rest remained optically transparent and colorless.

Both pre- and post-heated **M3** show a broad increase in absorbance across the visible and NIR region with increasing positive voltages applied with onset at +1.2 V, accompanied by the collapse of UV-A absorption bands (Figure 3c). While both pre- and post-heated **M3** show the emergence of a new visible peak at ca. 505 nm, the λ_abs_ values of the new absorption band in the NIR region are ca. 950 and 1000 nm, respectively, which slightly intensify and blue-shift to 900 and 960 nm, respectively, with applied voltages increasing beyond +1.9 and +2.0 V, respectively, to +2.4 V. Meanwhile, pre-heated **M4** shows two new distinct absorption bands at λ_abs_ of 585 and 890 nm, respectively, which emerges at +1.2 V and peaks in intensity at +1.8 V. This is accompanied by the collapse of the neutral 366 nm absorption peak. Further increase in applied voltages to +2.4 V, however, led to the broadening of the absorption band across the visible and NIR region. Similarly, increasing applied voltages on heated **M4** ECD resulted in a gradual broad increase in absorbance across the visible and NIR region with λ_abs_ at 1020 nm (Figure 3d). The electro-oxidation of both pre-heated **M3** and **M4** led to the change from slight yellow to a grayish tint while maintaining a high level of optical transparency. Their heated counterparts, however, showed modest changes in both optical transparency and tint color before and after electro-oxidation.

Chronoabsorptometry studies for ECDs fabricated using heated **M1**–**M4** were further performed by switching at ± 2.0 V at different time intervals (Δ*t*), and the transmittance changes (Δ%T) for a few cycles at 715, 930, 505, and 875 nm were recorded for **M1**, **M2**, **M3** and **M4**, respectively (Figure S5, ESI). It was found that EC switching at Δ*t* of 40 s (i.e., +2.0 V for 40 s, followed by −2.0 V for 40 s) only partially oxidized **M1**, as shown in the trends and shapes of Δ%T curves. The average Δ%T for **M1** at Δ*t* = 40 s was found to be a modest 9.7%, which decreased to 9.6%, 6.5%, 3.8%, and 2.3% as Δ*t* reduced from 40 to 30, 20, 10, and 5 s. The oxidation of **M2** and **M3** was more complete at Δ*t* = 40 s, and the Δ%T slightly improved to 11% and 13%, respectively. Their bleaching response times (τ_B_) upon electro-oxidation were rather consistent at 28.5 s, whereas the coloration response time (τ_c_) of the former upon electro-reduction was half that of the latter (6.6 vs. 14.7 s, respectively). Meanwhile, **M4** exhibited a Δ%T of 17% at 40 s Δ*t*, which decreased to 14.4% and 8.8% as Δ*t* decreased to 30 and 20 s, respectively. The modest Δ%T recorded for all four derivatives is reflected in the high optical transparency retained and the relatively modest intensity of new absorption bands emerging after electro-oxidation.

EC switching stability studies were further performed for **M2**, **M3**, and **M4** at applied voltages of ± 2.0 V and Δ*t* of 30 s at the same wavelengths of interest (Figures S6–S8, ESI). The Δ%T of **M2** remained relatively constant at ~10.5% for the first 100 cycles, but had decreased to ~7.6% by the 200th cycle. On the other hand, Δ%T of **M3** gradually decreased from 10.6% at the beginning to 8.3% and then 7.2% by the 50th and 100th cycle, respectively. For **M4**, the shape of the Δ%T profile changed as both the maximum and minimum %T decreased, with the latter decreasing more than the former, within the first 100 cycles, which likely indicates a chemical side reaction taking place. The Δ%T of the following 100 cycles, however, became relatively steady, with maximum and minimum %Ts between 82% and 66% and Δ%T decreasing from 15.4% to 13.5% from the 101st to 200th cycle, respectively.

**Figure 4:**
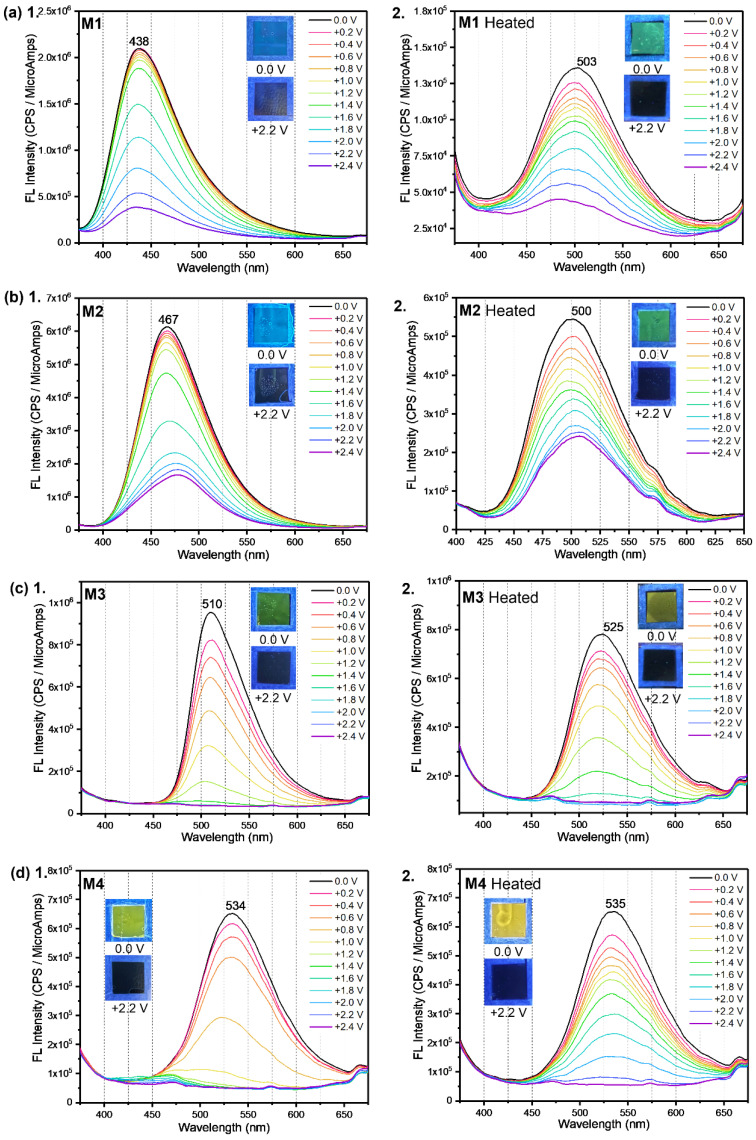
EFC spectroelectrochemistry of ECDs fabricated from (**a**) **M1**, (**b**) **M2**, (**c**) **M3**, and (**d**) **M4**. Insert: photos showing fluorescence of the respective ECDs at 0.0 and +2.2 V.

We next investigated the EFC properties of the four derivatives by monitoring the photoluminescence spectra changes in response to increasing applied positive voltages (Figure 4). Although **M1** and **M2** exhibited mild to moderate ACQ behavior, their thin films still revealed blue fluorescence with λ_FL_ at 438 and 467 nm, respectively. These were red-shifted when **M1** and **M2** were heated to induce cross-linking, where both heated derivatives revealed blue–green fluorescence with λ_FL_ at 503 and 500 nm, respectively. As applied voltages were increased from 0.0 to +2.4 V, the fluorescence of pre- and post-heated **M1** and **M2** was gradually quenched as they were electro-oxidized into radical cationic states. On the other hand, pre-heated **M3** and **M4** revealed bright greenish-yellow fluorescence with λ_FL_ at 510 and 534 nm, respectively, which upon heating exhibited bright yellow fluorescence with redshifted λ_FL_ of 525 and 535 nm, respectively. The application of positive voltage caused efficient fluorescence quenching for all four ECDs due to electro-oxidation, with the fluorescence of pre-heated **M3** and **M4** being completely quenched at +1.6 and +1.4 V, respectively, and that of post-heated **M3** and **M4** at slightly higher voltages of +2.0 and +2.4 V, respectively.

The EFC switching properties of post-heated derivatives at their respective λ_FL_ values were further evaluated. By applying ±2.0 V at Δ*t* of 30 s, it was found that upon successive electro-oxidation, the fluorescence of **M1**–**M3** barely recovered to its initial intensities when negative voltages were applied, whereas while **M4** showed better switching reversibility, fluorescence intensity changes were modest with an I_OFF/ON_ contrast ratio of only 1.4 (Figure S9, ESI). Prolonging or reducing the Δ*t* for **M2** did not improve I_OFF/ON_ either (Figure S10, ESI). We then considered reversing the EFC switching regime by first applying negative voltage followed by positive voltage, with the former at a higher magnitude than the latter. Switching between −2.2 and +1.8 V at 30 s Δ*t*, **M3** achieved a significantly improved I_OFF/ON_ of 9.5, but decreased to 8.4 by the fifth cycle, as cycling was still not quite reversible, with the recovered maximum fluorescence intensity reducing every successive cycle. However, when the applied positive voltage was reduced to +1.6 V, we found that the cycling reversibility had clearly improved, with an I_OFF/ON_ of 6.0 (Figure S11a, ESI). **M4** showed better switching reversibility when switching both between −2.2/+1.6 V or −2.2/+1.8 V at 30 s Δ*t*, with both having an I_OFF/ON_ of approximately 4.8 (Figure S11b, ESI).

**Figure 5:**
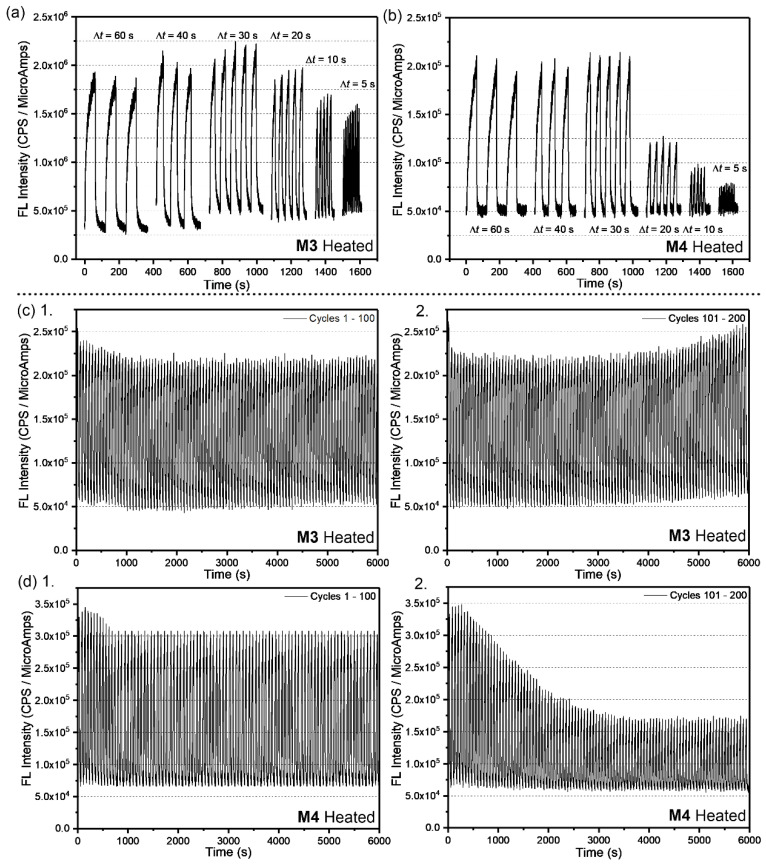
EFC switching of post-heated (**a**) **M3** at −2.2/+1.6 V and (**b**) **M4** at −2.2/+1.8 V at different Δ*t*s of 60, 40, 30, 20, 10, and 5 s. EFC cycling stability studies of (**c**) **M3** and (**d**) **M4**, switching between 2.2/+1.6 V and −2.2/+1.8 V, respectively, at 30 s Δ*t*.

Further EFC switching studies were performed at different Δ*t*s for post-heated **M3** and **M4** (Figure 5a,b). Increasing Δ*t* from 30 to 40 and 60 s led to slight improvement in I_OFF/ON_ for **M3** to 6.3 and 7.0, respectively, whereas I_OFF/ON_ remained relatively constant for **M4**. On the other hand, I_OFF/ON_ for both derivatives decreased when Δ*t* was reduced. These were more drastic for **M4** with I_OFF/ON_ of 2.8, 2.2 and 1.7 at Δ*t* = 20, 10, and 5 s, respectively, whereas those for **M3** were higher at 4.8, 3.9, and 3.2, respectively. Meanwhile, the response times for **M3** and **M4** were determined to be 16.5 and 8.1 s, respectively, for the “switching off”, but much slower at 50.6 and 55.0 s, respectively, for the “turning on” of fluorescence, reflecting the relative preferences of both electroactive derivatives to be in the oxidized states.

Finally, the long-term EFC cycling stability of heated **M3** and **M4** was evaluated under the previous switching conditions at Δ*t* of 30 s. As shown in Figure 5c, the cycling stability of **M3** was fairly stable over the 200 switching cycles with not much loss in I_OFF/ON_. On the other hand, **M4** exhibited relatively poorer cycling stability, where its I_OFF/ON_ remained relatively stable for the first 100 cycles, but started to deteriorate during the subsequent 100 cycles, with the absolute difference in fluorescence intensity between “off” and “on” states to be almost half that at the beginning (Figure 5d).

## 3. Materials and Methods

### 3.1. Materials and Instrumentation

All chemicals, reagents, and solvents were purchased from commercial vendors, e.g., Sigma Aldrich, TCI, and used without purification unless otherwise stated. Compounds synthesized were purified with column chromatography over silica gel grade 60 (Merck, Singapore) 0.040–0.063 mm, 230–400 mesh). ITO-coated glass substrates (15 Ω/sq; 30 mm × 35 mm × 1.1 mm) were purchased from Xin Yan Technology Ltd. (Hong Kong, China), and were washed generously with deionized water and acetone, followed by ozone surface treatment at 100 °C for 10 min before use. ^1^H nuclear magnetic resonance (NMR) spectra of compounds were recorded at 25 °C in deuterated solvent (purchased from Cambridge Isotopes Laboratories (Singapore)) using a Jeol 500 MHz NMR spectrometer. Chemical shifts (δ) are expressed with a positive sign, in parts per million (ppm), relative to residual solvent signals as reference. High-resolution mass spectrometry (HRMS) of synthesized compounds was performed using an Agilent 7200 GC-QTOF mass spectrometry system (Agilent, Santa Clara, CA, USA). Fourier-transform infrared (FTIR) spectrometry was performed with a Perkin Elmer Spectrum 2000 FTIR spectrometer in KBr pellets (Perkin Elmer, Waltham, MA, USA). Ultraviolet-visible (UV-vis) absorption spectrophotometry, EC spectroelectrochemistry, chronoabsorptometry, and EC stability studies were performed using a Shimadzu UV3600 UV-vis–NIR spectrophotometer (Shimadzu, Kyoto, Japan). Photoluminescence and EFC studies were performed with a Horiba Jobin Yvon Fluorolog^®^ (Horiba: Kyoto, Japan) spectrofluorometer. Solid-state photoluminescence quantum yields were measured using an integrating sphere with a laser excitation source of 405 nm. Electrochemical studies were performed using a Metrohm Autolab PGSTAT128N potentiostat/galvanostat (Metrohm Autolab BV, Utrecht, The Netherlands). Scanning electron microscopy (SEM) of thin films was performed using a Jeol JSM-6700F SEM (Jeol, Tokyo, Japan).

### 3.2. Synthesis of Materials

Tris(4-vinylphenyl)amine (**M1**). Tris-(4-bromophenyl)amine (241 mg, 0.5 mmol), 4,4,5,5-tetramethyl-2-vinyl-1,3,2-dioxaborolane (254 mg, 1.65 mmol), and Pd(PPh_3_)_4_ (87 mg, 0.075 mmol) were added to a flame-dried 2-neck round-bottomed flask (RBF) fitted with a condenser. The setup was evacuated and back-filled with argon gas 3 times, and aqueous potassium carbonate solution (1 M, 2.5 mL), ethanol (2.5 mL), and toluene (10 mL) were added. The reaction mixture was thoroughly degassed by purging with argon gas for 30 min with stirring, then allowed to stir at 95 °C for 18 h. Thereafter, the reaction mixture was cooled to room temperature and quenched with aqueous sodium hydrogen carbonate solution. The mixture was extracted with chloroform 3 times and the combined organic fractions were dried over magnesium sulfate. The crude product was concentrated in vacuo and purified via column chromatography using DCM–hexane (1:4 *v*/*v*) to yield a white solid product (113 mg, 70% yield). ^1^H NMR (500 MHz, CDCl_3_, δ): 5.17 (d, *J* = 11.0 Hz, 3H), 5.65 (d, *J* = 17.5 Hz, 3H), 6.67 (dd, *J* = 11.0 Hz, 17.5 Hz, 3H), 7.04 (d, *J* = 8.5 Hz, 6H), 7.30 (d, *J* = 8.5 Hz, 6H). ^13^C NMR (100 MHz, CDCl_3_, δ): 112.42, 124.04, 127.11, 132.34, 136.14, 146.95. HRMS (ESI, *m*/*z*): [M]^+^ calculated for C_24_H_21_N, 323.1674; measured, 323.16751.

Tris(4′-vinyl-[1,1′-biphenyl]-4-yl)amine (**M2**). **M2** was synthesized via the same procedure as **M1**, involving Suzuki cross-coupling between tris-(4-bromophenyl)amine (241 mg, 0.5 mmol) and 4-vinylphenylboronic acid (245 mg, 1.65 mmol). The concentrated crude product was purified via column chromatography using DCM–hexane (1:1 *v*/*v*) to yield an off-white solid product (244 mg, 89% yield). ^1^H NMR (500 MHz, CDCl_3_, δ): 5.27 (d, *J* = 11.0 Hz, 3H), 5.79 (d, *J* = 17.5 Hz, 3H), 6.76 (dd, *J* = 11.0, 17.5 Hz, 3H), 7.23 (d, *J* = 8.0 Hz, 6H), 7.48 (d, *J* = 8.0 Hz, 6H), 7.53 (d, *J* = 8.0 Hz, 6H), 7.57 (d, *J* = 8.0 Hz, 6H). ^13^C NMR (100 MHz, CDCl_3_, δ): 113.68, 124.42, 126.66, 126.71, 127.71, 135,14, 136.22, 136.41, 139.91, 146.80. HRMS (ESI, *m*/*z*): [M]^+^ calculated for C_42_H_33_N, 551.2613; measured, 551.26182.

Bis(4-vinylphenyl)amine (**1**). Compound **1** was synthesized via the same procedure as **M1**, involving Suzuki cross-coupling between bis(4-bromophenyl)amine (980 mg, 3 mmol) and 4,4,5,5-tetramethyl-2-vinyl-1,3,2-dioxaborolane (1.02 g, 6.6 mmol). The concentrated crude product was purified via column chromatography using DCM–hexane (1:1 *v*/*v*) to yield a white crystalline solid product (300 mg, 45% Yield). ^1^H NMR (500 MHz, CDCl_3_, δ): 5.13 (d, *J* = 11.0 Hz, 2H), 5.63 (d, *J* = 17.5 Hz, 2H), 5.80 (s, broad, 1H), 6.67 (dd, *J* = 11.0, 17.5 Hz, 2H), 7.03 (d, *J* = 10.0 Hz, 4H), 7.33 (d, *J* = 10.0 Hz, 4H). ^13^C NMR (100 MHz, CD_2_Cl_2_, δ): 111.39, 117.87, 127.63, 131.00, 136.67, 142.91. HRMS (ESI, *m*/*z*): [M]^+^ calculated for C_16_H_15_N, 221.12045; measured, 221.12036. 

Bis(4′-vinyl-[1,1′-biphenyl]-4-yl)amine (**2**). Compound **2** was synthesized via the same procedure as **M1**, involving Suzuki cross-coupling between bis(4-bromophenyl)amine (980 mg, 3 mmol) and 4-vinylphenylboronic acid (976 g, 6.6 mmol). The concentrated crude product was purified via column chromatography using CHCl_3_–hexane (0:1 to 1:1 to 1:0 *v*/*v*), and subsequently washed with hexane and methanol to yield a an off-white solid product (450 mg, 40% yield). ^1^H NMR (500 MHz, THF-d8, δ): 5.18 (d, *J* = 10.9 Hz, 2H), 5.77 (d, *J* = 17.6 Hz, 2H), 6.74 (dd, *J* = 10.9, 17.6 Hz, 2H), 7.18 (d, *J* = 8.7 Hz, 4H), 7.46 (d, *J* = 8.4 Hz, 4H), 7.55 (m, 8H), 7.62 (s, broad, 1H). ^13^C NMR (100 MHz, THF-d8, δ): 113.31, 118.41, 118.84, 127.09, 127.58, 128.38, 133.37, 136.87, 137.81, 141.49, 144.28. HRMS (ESI, *m*/*z*): [M]^+^ calculated for C_28_H_23_N, 373.18305; measured, 373.18267.

4,4′,4″,4‴-(ethene-1,1,2,2-tetrayl)tetrakis(*N*,*N*-bis(4-vinylphenyl)aniline) (**M3**). 1,1,2,2-tetrakis(4-bromophenyl)ethene (324 mg, 0.5 mmol), compound **1** (553 mg, 2.5 mmol), sodium tert-butoxide (384 mg, 4 mmol) and palladium(II) acetate (46 mg, 0.05 mg) were added to a flame-dried 2-neck RBF fitted with a condenser. The setup was evacuated and back-filled with argon gas 3 times. Thereafter, tri-tert-butylphosphine (10 wt% in hexane, 0.45 mL, 0.15 mmol) and anhydrous toluene (30 mL) were added. The reaction mixture was allowed to stir at reflux for 18 h. On cooling to room temperature, the mixture was filtered and the filtrate was concentrated in vacuo. The crude product was purified via column chromatography using chloroform–hexane (1:1 *v*/*v*) as eluent to afford a bright-yellow solid product (335 mg, 55% yield). ^1^H NMR (500 MHz, THF-d8, δ): 5.09 (d, *J* = 11.0 Hz, 8H), 5.62 (d, *J* = 17.6 Hz, 8H), 6.64 (dd, *J* = 11.0, 17.6 Hz, 8H), 6.88 (d, *J* = 8.6 Hz, 8H), 7.01 (d, *J* = 8.6 Hz, 24H), 7.30 (d, *J* = 8.2 Hz, 16H). ^13^C NMR (100 MHz, THF-d8, δ): 112.68, 124.19, 125.14, 128.30, 133.54, 133.76, 137.54, 139.87, 141.24, 146.98, 148.31. HRMS (ESI, *m*/*z*): [M]^+^ calculated for C_90_H_72_N_4_, 1208.5757; measured, 1208.57044.

*N*,*N*′,*N*″,*N*‴-(ethene-1,1,2,2-tetrayltetrakis(benzene-4,1-diyl))tetrakis(4′-vinyl-N-(4′-vinyl-[1,1′-biphenyl]-4-yl)-[1,1′-biphenyl]-4-amine) (**M4**). **M4** was synthesized via the same procedure as **M3**, involving a Buchwald–Hartwig amination reaction between 1,1,2,2-tetrakis(4-bromophenyl)ethene (324 mg, 0.5 mmol) and compound **2** (935 mg, 2.5 mmol). The crude product was purified via column chromatography using chloroform–hexane (1:1 to 1:0 *v*/*v*) as eluent to afford a bright-yellow solid product (364 mg, 40% yield). ^1^H NMR (500 MHz, THF-d8, δ): 5.19 (d, *J* = 10.9 Hz, 8H), 5.76 (d, *J* = 17.5 Hz, 8H), 6.71 (dd, *J* = 10.9, 17.5 Hz, 8H), 7.01 (d, *J* = 8.3 Hz, 8H), 7.11 (d, *J* = 8.6 Hz, 8H), 7.18 (d, *J* = 8.0 Hz, 16H), 7.41 (d, *J* = 8.0 Hz, 16H), 7.52 (d, *J* = 8.3 Hz, 16H), 7.55 (d, *J* = 8.0 Hz, 16H). ^13^C NMR (100 MHz, THF-d8, δ): 113.83, 124.41, 125.51, 127.59, 127.78, 128.73, 129.21, 129.97, 133.65, 136.31, 137.49, 137.84, 141.04, 147.16, 148.12. HRMS (ESI, *m*/*z*): [M]^+^ calculated for C_138_H_104_N_4_, 1816.8256; measured, 1816.8229.

### 3.3. Cyclic Voltammetry

Cyclic voltammetry (CV) of **M1**–**M4** was performed by dissolving each derivative in solution electrolyte containing tetrabutylammonium hexafluorophosphate (~0.1 M in dichloromethane), with the use of an Ag/AgCl reference electrode (0.197 V vs. SHE), a Pt wire counter electrode, and a glassy carbon working electrode. The solutions were thoroughly degassed, and analysis scans were run at a scan rate of 50 mV/s within the range of +/−1.5 V. The results were then calibrated against ferrocene as reference. CVs on fabricated device were performed by connecting the working electrode to a substrate coated with materials and a counter electrode over the adjacent substrate, with a reference electrode connected behind that of the counter electrode. Scans were then performed at a scan rate of 50 mV/s.

### 3.4. Fabrication of ECDs

A quantity of 150 μL of **M1**–**M4** solutions (10 mg/mL in chloroform and filtered over 0.45 μm PVDF filter frit) was spin-coated over the ITO surface of cleaned ITO–glass substrates at 500 rpm for 30 s. Excess edges of coated substrates were then wiped off using a chloroform-dampened cotton bud to obtain a 2 cm × 2 cm active area. To promote cross-linking, the coated substrates were heated at 215 °C for 30 min on a hotplate in the glovebox under a N_2_ environment. On another set of cleaned ITO–glass substrates, 275 μL of gel was pipetted onto the ITO surface into a demarcated 2 cm × 2 cm area blocked out using double-sided adhesive tapes. It was then left to rest under ambient conditions for 20 min. The gel electrolyte was prepared by stirring 0.512 g lithium perchlorate, 6.65 mL propylene carbonate, and 2.8 g poly(methylmethacrylate) (M_W_ 120 kDa) in 28 mL of anhydrous acetonitrile. To assemble the ECD, (unheated or heated) substrates containing **M1**–**M4** were sandwiched with the electrolyte-deposited substrates, with an uncontacted edge of 0.5 cm on both sides for electrical contact.

### 3.5. Spectroelectrochemistry and Chronoabsorptometry Studies

For spectroelectrochemistry, the absorption and photoluminescence spectra of ECD were recorded with a UV-vis–NIR absorption spectrophotometer and photoluminescence spectrophotometer, respectively, while the two uncontacted edges of the ECDs were connected to the potentiostat, which controls the magnitude and duration of applied voltage. For the former, an assembled “blank” ECD containing no deposited materials was used as reference. For chronoabsorptometry and EFC switching studies, the transmittance and photoluminescence intensity, respectively, at particular wavelengths of interest were recorded over time using the spectrophotometer in kinetics mode, while applied voltages were modulated through the potentiostat.

## 4. Conclusions

In summary, we prepared four derivatives, **M1**–**M4**, with multiple terminal styryl groups to promote cross-linking when heated. The derivatives all contain electroactive TPA moieties to enable EC and EFC switching, with **M3** and **M4** also adopting a TPE scaffold to endow AIE properties. ECDs were then fabricated to study the effects of heat-induced cross-linking on the EC and EFC properties. It was worth noting that while heating-caused cross-linking led to blueshift in absorption profiles of **M1**–**M4**, their fluorescence spectra were redshifted.

EC studies showed that both pre- and post-heated **M1**–**M4** maintained a high level of optical transparency before and after application of positive voltages due to modest changes in the visible region of the absorption spectra. Meanwhile, EFC studies showed that the derivatives revealed quenching of blue to yellow thin-film fluorescence with increasing positive voltages applied. While heated **M1** and **M2** revealed modest cycling reversibility and I_OFF/ON_ contrast ratios, respectively, **M3** and **M4** achieved better reversibility with switching regimes of −2.2/+1.6 and −2.2/+1.8 V, respectively. In particular, **M3** achieved an I_OFF/ON_ ratio of up to 7.0 when switching at Δ*t* of 60 s. Further EFC long-term cycling stability studies performed at the same respective voltages at Δ*t* of 30 s, showed that **M3** outperformed **M4** within 200 switching cycles. The derivatives would therefore serve as promising EFC materials with tunable fluorescence colors with high optical transparency. More importantly, this work also demonstrated the viability of developing styryl-functionalized electroactive derivatives that are heat-induced cross-linkable via a simple cross-coupling method in future designs of better-performing EC and EFC materials.

## Figures and Tables

**Table 1:** Optical and electrochemical properties of **M1**–**M4**.

	λ_abs_ ^Soln.^ (nm) ^a^	λ_abs_ ^Film^ (nm) ^a^	ε (M^−1^ cm^−1^) ^b^	λ_onset_ ^Soln.^ (nm) ^c^	E_g_ ^opt.^ (eV) ^d^	λ_FL_ ^Soln.^ (nm) ^e^	λ_FL_ ^Film^ (nm) ^e^	Φ_FL_^Soln.^ (%) ^f^	Φ_FL_^Solid^ (%) ^g^	E_HOMO_ (eV) ^h^	E_LUMO_ (eV) ^i^
**M1**	350	339	51 868	385	3.22	405	438	12.3	6.7	−4.94	−1.72
**M2**	359	361	36 079	406	3.05	435	467	13.6	5.4	−5.03	−1.98
**M3**	353	362	134 125	457	2.71	553	510	0.08	26.3	−4.92	−2.21
**M4**	361	366	155 573	462	2.68	430, 553	533	0.15	26.6	−4.97	−2.29

^a^ Absorption maximum wavelength; ^b^ molar extinction coefficient; ^c^ absorption onset wavelength; ^d^ optical bandgap = 1240/λ_onset_; ^e^ fluorescence maximum wavelength; ^f^ fluorescence quantum yield measured in reference to quinine sulfate in 0.5 M H_2_SO_4_ (**Φ**_FL_ = 54%), with measurements subjected to ±10% error; ^g^ fluorescence quantum yield in solid state; ^h^ HOMO energy level determined by cyclic voltammetry; ^i^ LUMO energy level = E_HOMO_ + E_g_ ^opt.^.

## Data Availability

The original contributions presented in the study are included in the article/Supplementary Materials, further inquiries can be directed to the corresponding author.

## Supplementary Materials

The following supporting information can be downloaded at https://www.mdpi.com/article/10.3390/molecules29102340/s1.
