# Supplementary material for: Electrofluorochromic Switching of Heat-Induced Cross-Linkable Multi-Styryl-Terminated Triphenylamine and Tetraphenylethylene Derivatives
Source: Molecules. 2024 May 16;29(10):2340. doi: 10.3390/molecules29102340 (PMC11123742; doi:10.3390/molecules29102340)
Supplement: Supplementary file 1 [file molecules-29-02340-s001.zip › molecules-2966025-supplementary.pdf]

Supporting Information

Electrofluorochromic Switching of Heat-induced Cross-linkable Multi Styryl-terminated Triphenylamine and Tetraphenylethylene Derivatives

Kang Le Osmund Chin, [a] Pin Jin Ong, [b] Qiang Zhu, [a, b] Jianwei Xu, [*, a, c] Ming Hui Chua [*, a]

a. *Institute of Sustainability for Chemicals, Energy and Environment (ISCE^2), Agency for Science, Technology and Research (A*STAR), Singapore. 1 Pesek Road, Jurong Island, Singapore 627833*

b. *Institute of Materials Research and Engineering (IMRE), Agency for Science, Technology and Research (A*STAR), Singapore. 2 Fusionopolis Way, Innovis, #08-03, Singapore 138634*

c. *Department of Chemistry, National University of Singapore (NUS), Singapore. 3 Science Drive 3, Singapore 117543*

* Corresponding: M. H. Chua (chua_ming_hui@isce2.a-star.edu.sg); J. Xu (xu_jianwei@isce2.a-star.edu.sg)

CONTENT

Figure	Figure Caption	Page
S1	FTIR spectra of **M1** – **M4** before and after heating	2
S2	Normalized absorption and fluorescence spectra of **M1** – **M4** in chloroform solutions	2
S3	Cyclic voltammograms of **M1** – **M4** in solution and in device forms.	3
S4	Normalized absorption spectra of **M1** – **M4** thin films before and after heating	3
S5	Chronoabsorptometry studies: Transmittance changes of heated **M1** – **M4** at 715, 930, 505, and 875 nm, successively, recorded upon switching at ± 2.0 V at different time intervals (Δ*t*)	4
S6	EC switching stability studies of **M2** at applied voltages of ± 2.0 V and Δ*t* of 30 s	4
S7	EC switching stability studies of **M3** at applied voltages of ± 2.0 V and Δ*t* of 30 s	5
S8	EC switching stability studies of **M4** at applied voltages of ± 2.0 V and Δ*t* of 30 s	5
S9	Fluorescence intensity changes of heated (a) **M1**, (b) **M2**, (c) **M3**, and (d) **M4** at 500, 500, 510, and 535 nm, respectively, with applied voltages of ±2.0 V at Δ*t* of 30 s	6
S10	Fluorescence intensity changes of heated **M2** at 500 nm with applied voltages of ±2.0 V at different Δ*t* s	6
S11	Fluorescence intensity changes of heated (a) **M3** and (b) **M4** at 510 and 535 nm, respectively, with applied voltages of -2.2 / +1.6 and -2.2 / +1.8 V, at Δ*t* of 30 s	7
**Table**	**Table Caption**	
S1	Fluorescence quantum yield (Φ_{FL}) of EFC devices	7
**ANNEX 1**: NMR Spectroscopy		8
**ANNEX 2**: High Resolution Mass Spectrometry (HRMS)		14

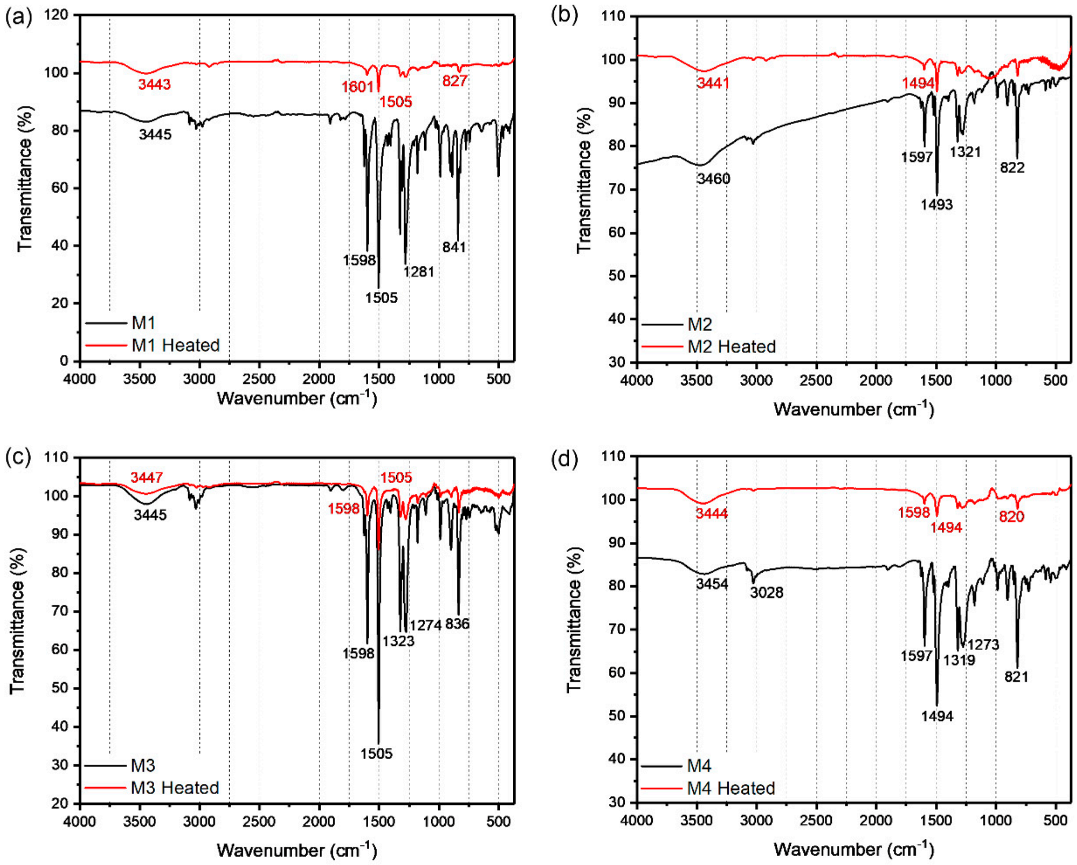

Figure S1. FTIR spectra of **M1** – **M4** before and after heating.

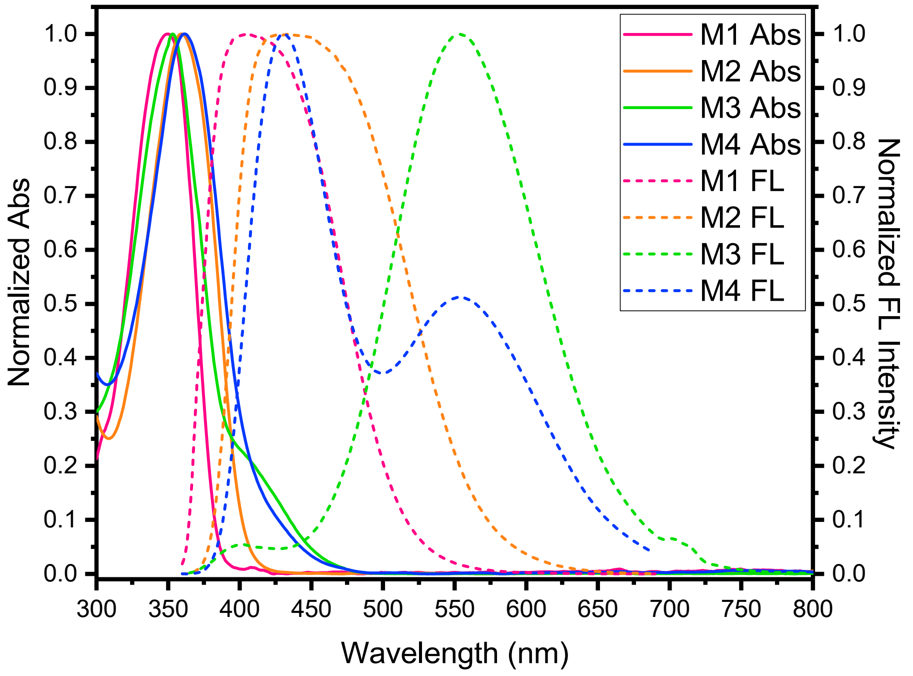

Figure S2. Normalized absorption and fluorescence spectra of **M1** – **M4** in chloroform solutions.

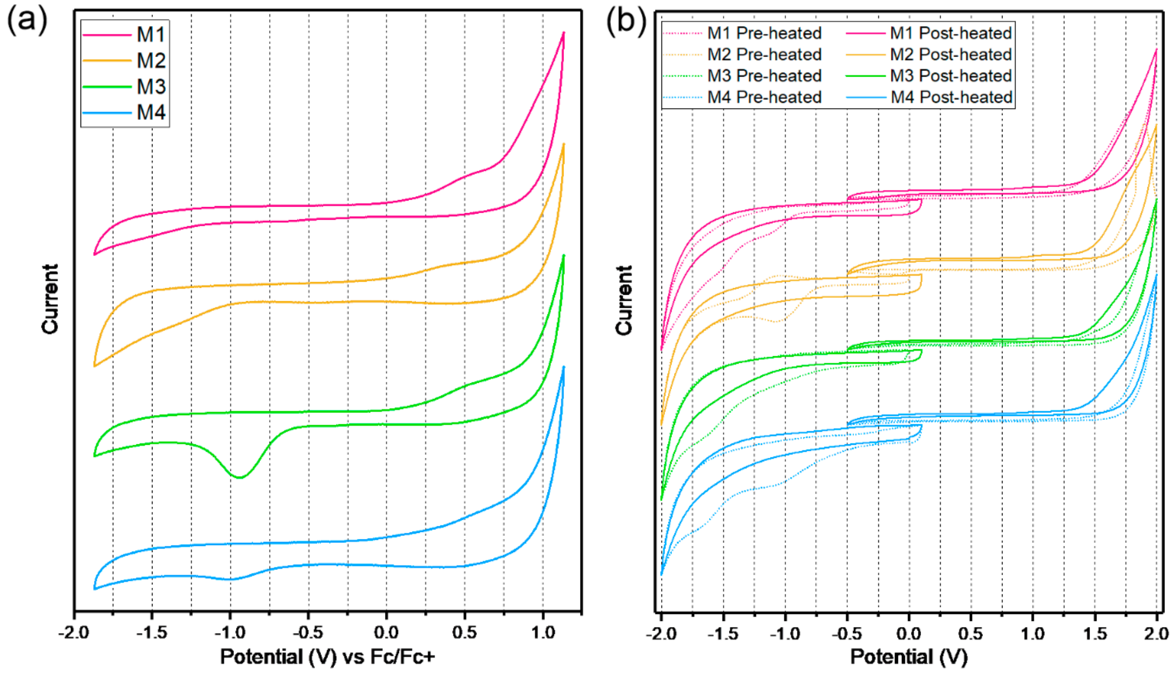

Figure S3. (a) Cyclic voltammograms of **M1** – **M4** in solution containing tetrabutylammonium hexafluorophosphate electrolyte (~0.1 M in DCM) calibrated against ferrocene reference. (b) Cyclic voltammograms of electrochromic devices (ECDs) containing pre- and post-heated **M1** – **M4**.

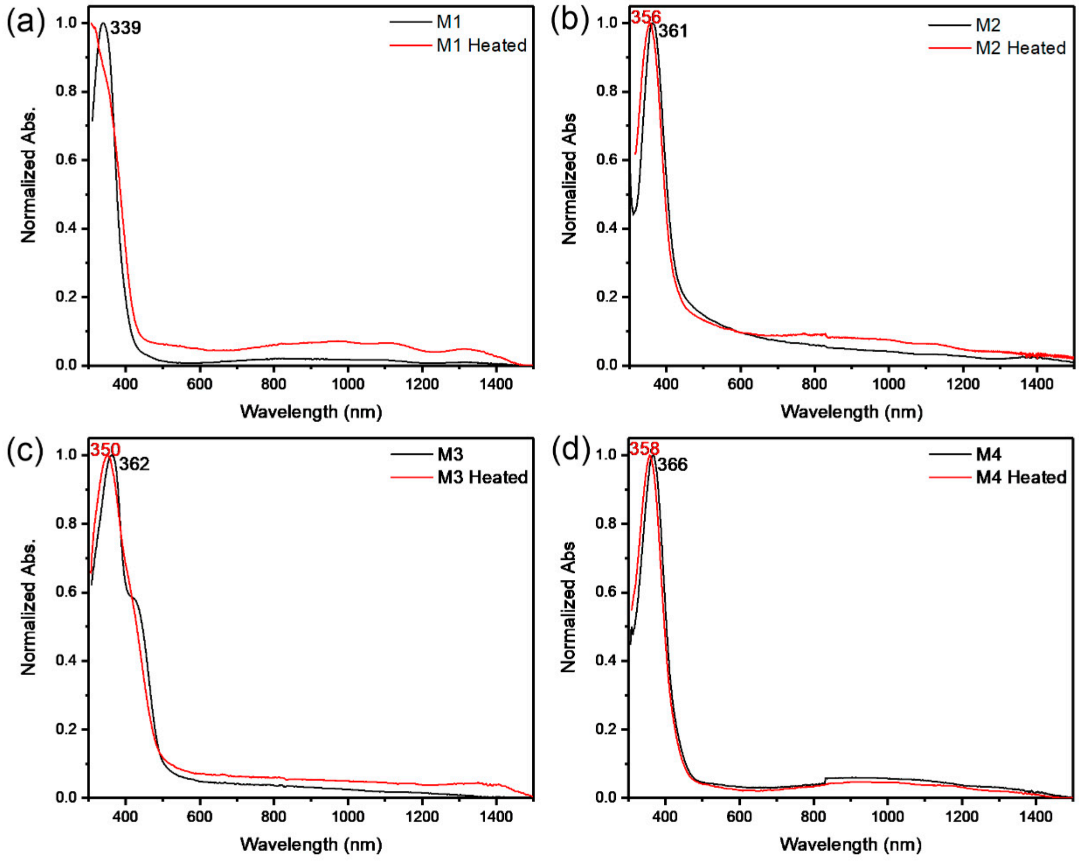

Figure S4. Normalized absorption spectra of **M1** – **M4** thin films before and after heating.

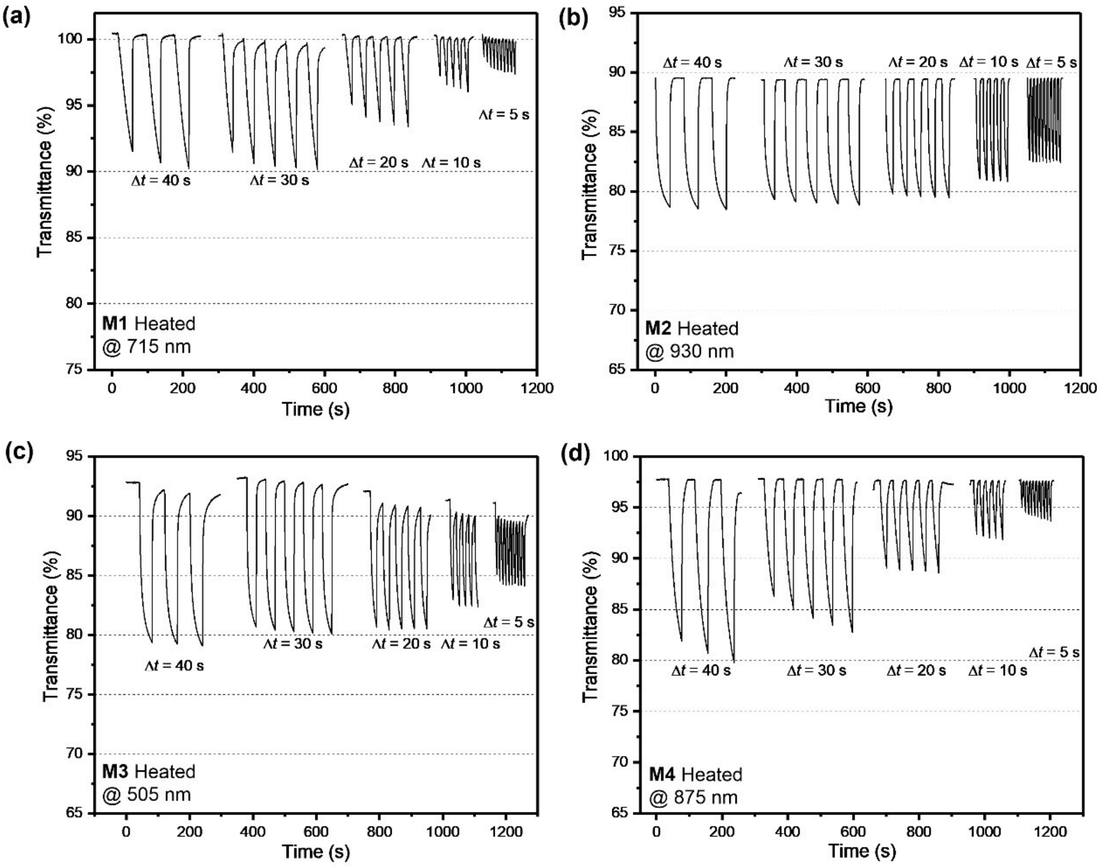

Figure S5. Chronoabsorptometry studies: Transmittance changes of heated **M1** – **M4** at 715, 930, 505, and 875 nm, successively, recorded upon switching at ± 2.0 V at different time intervals (Δ*t*).

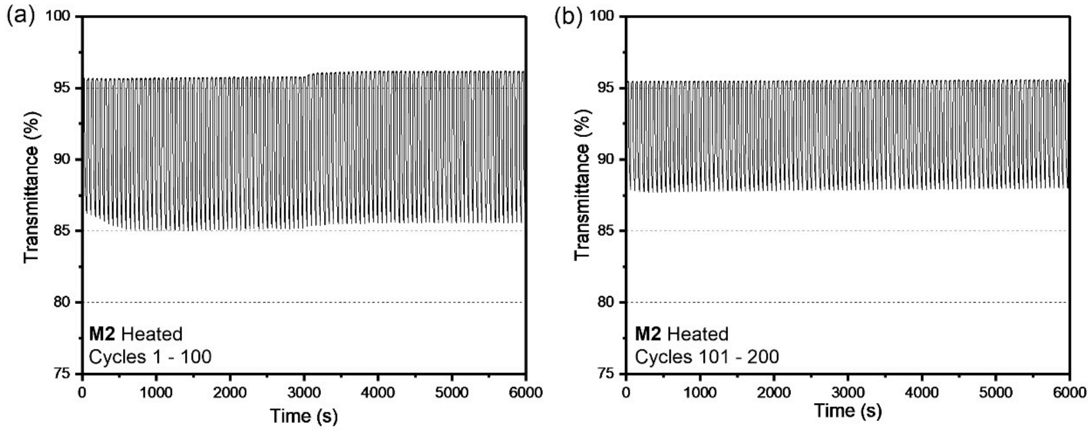

Figure S6. EC switching stability studies of **M2** at applied voltages of ± 2.0 V and Δ*t* of 30 s.

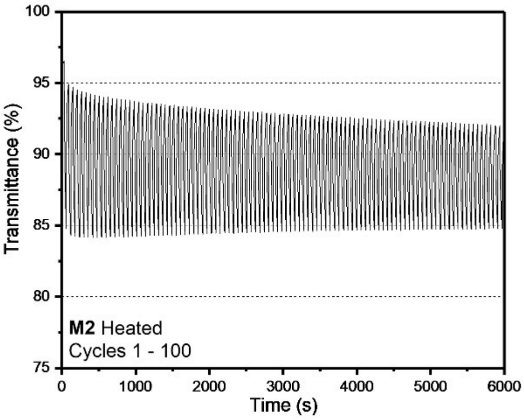

Figure S7. EC switching stability studies of **M3** at applied voltages of ± 2.0 V and Δt of 30 s.

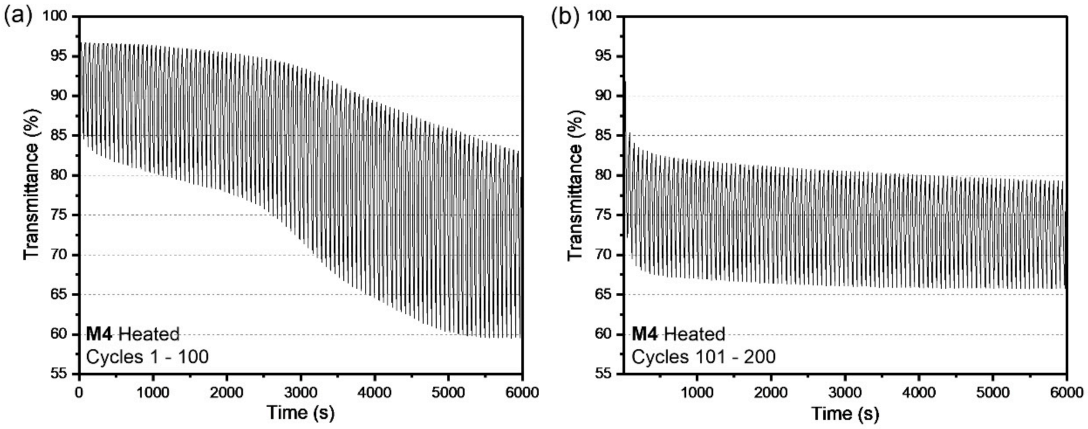

Figure S8. EC switching stability studies of **M4** at applied voltages of ± 2.0 V and Δt of 30 s.

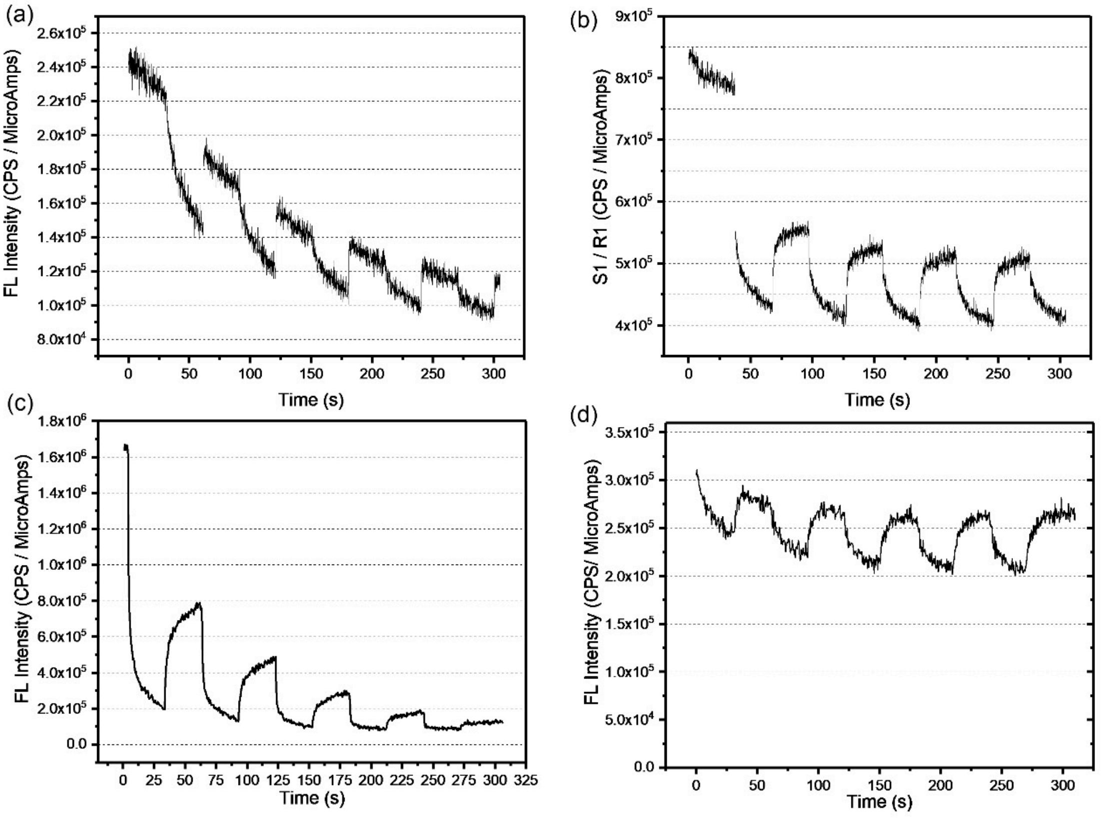

Figure S9. Fluorescence intensity changes of heated (a) **M1**, (b) **M2**, (c) **M3**, and (d) **M4** at 500, 500, 510, and 535 nm, respectively, with applied voltages of ±2.0 V at Δt of 30 s.

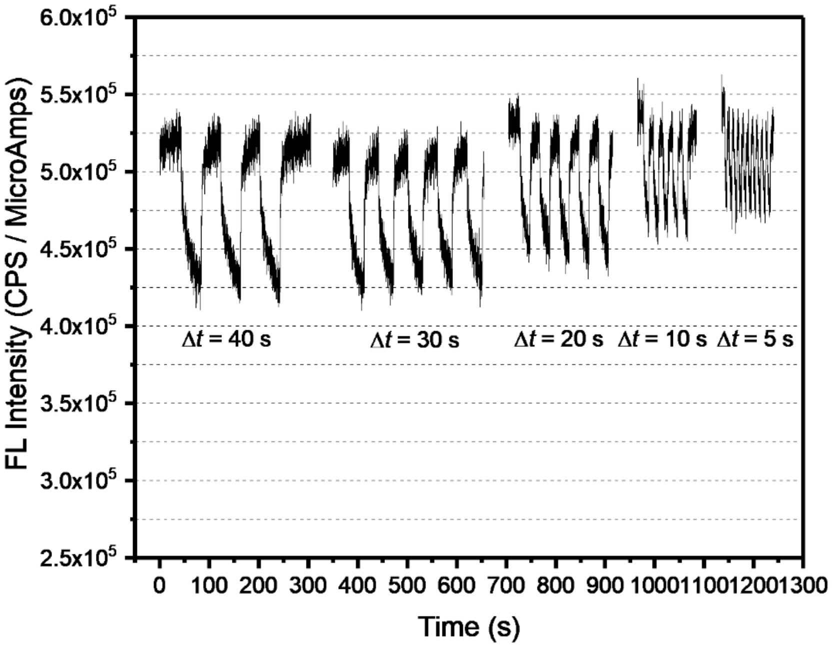

Figure S10. Fluorescence intensity changes of heated **M2** at 500 nm with applied voltages of ±2.0 V at different Δt s.

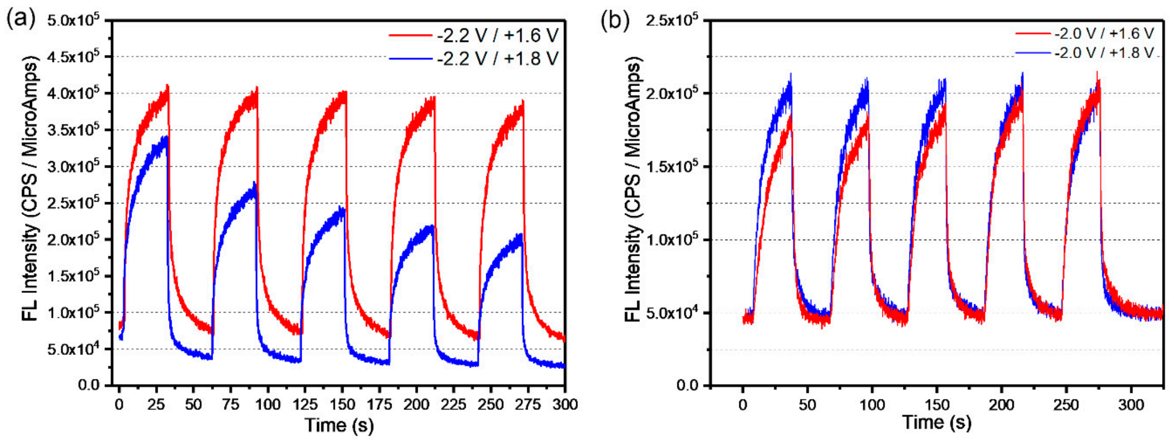

Figure S11. Fluorescence intensity changes of heated (a) **M3** and (b) **M4** at 510 and 535 nm, respectively, with applied voltages of -2.2 / +1.6 and -2.2 / +1.8 V, at Δ*t* of 30 s.

Table S1. Fluorescence quantum yield (Φ_{FL}) of EFC devices

Compound	Treatment of coated substrate	λ_{FL} (nm)	Φ_{FL} at 0.0 V (%)	Φ_{FL} at +2.0 V (%)
**M1**	Without Heating	438	5.08	0.92
	With Heating	503	3.25	0.12
**M2**	Without Heating	467	2.08	1.57
	With Heating	500	5.13	3.43
**M3**	Without Heating	510	3.34	2.39
	With Heating	525	1.39	0.03
**M4**	Without Heating	534	6.29	0.94
	With Heating	535	4.51	0.37

ANNEX 1: NMR Spectroscopy

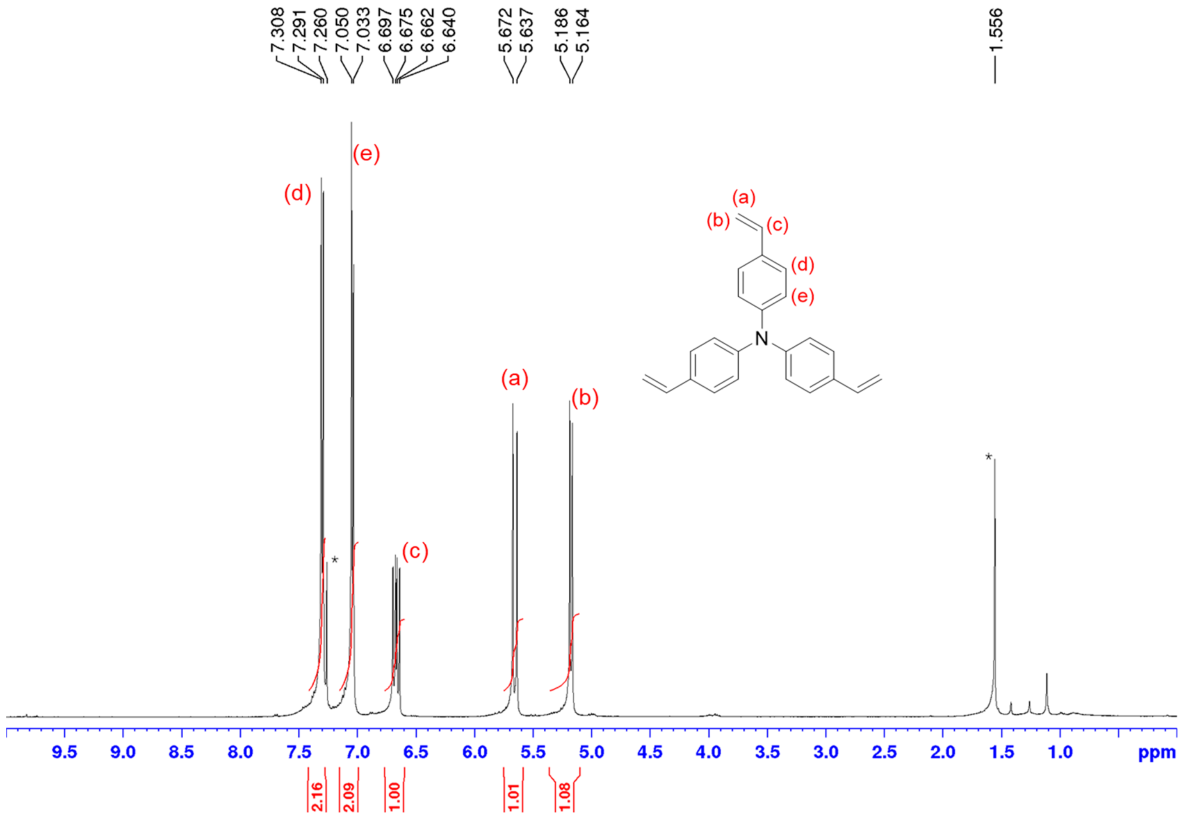

Figure S12. ^{1}H NMR of **M1** in $CDCl_3$

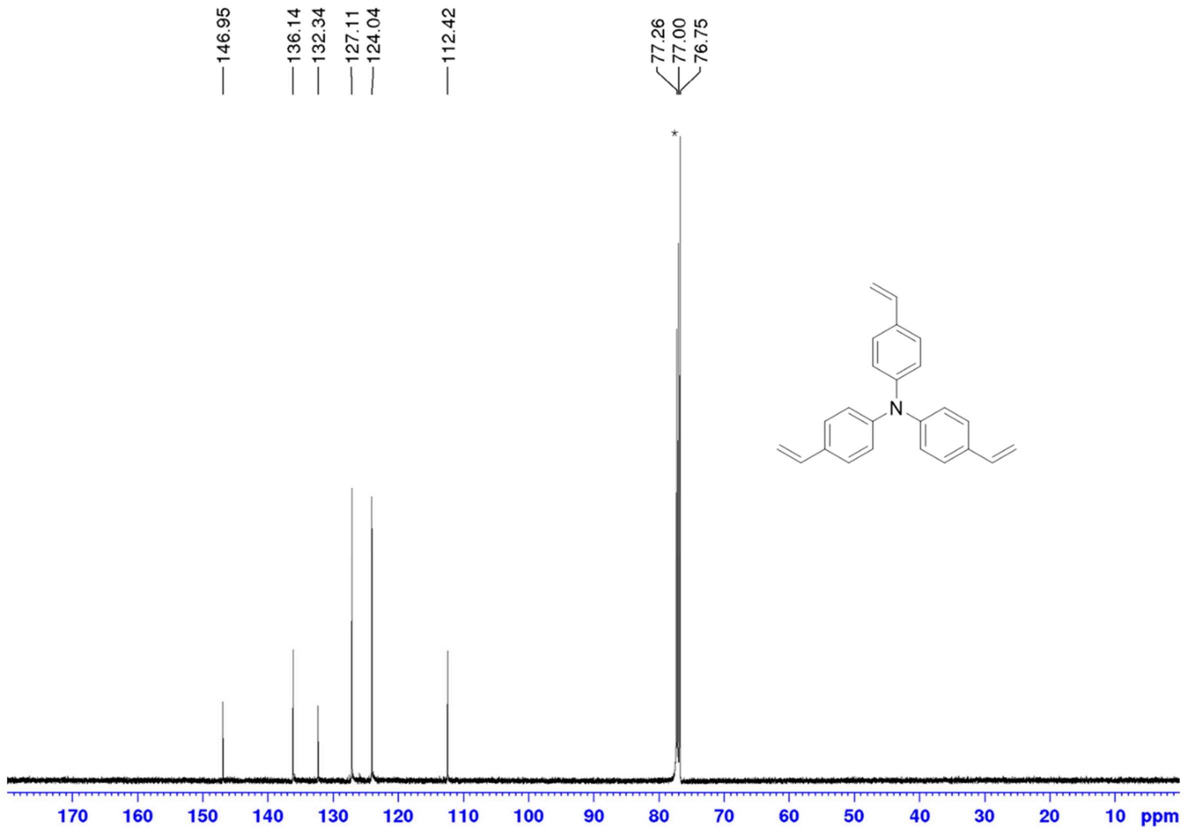

Figure S13. ^{13}C NMR of **M1** in $CDCl_3$

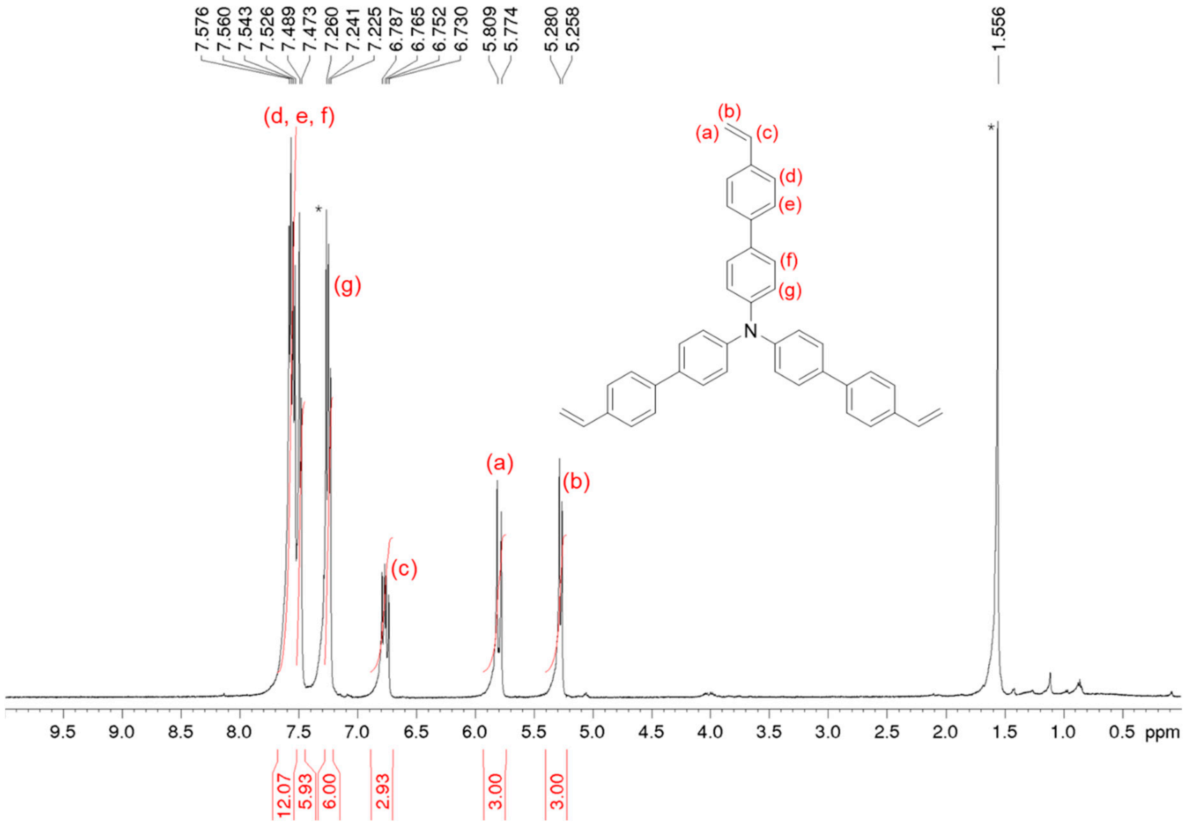

Figure S14. ^{1}H NMR of **M2** in $CDCl_3$

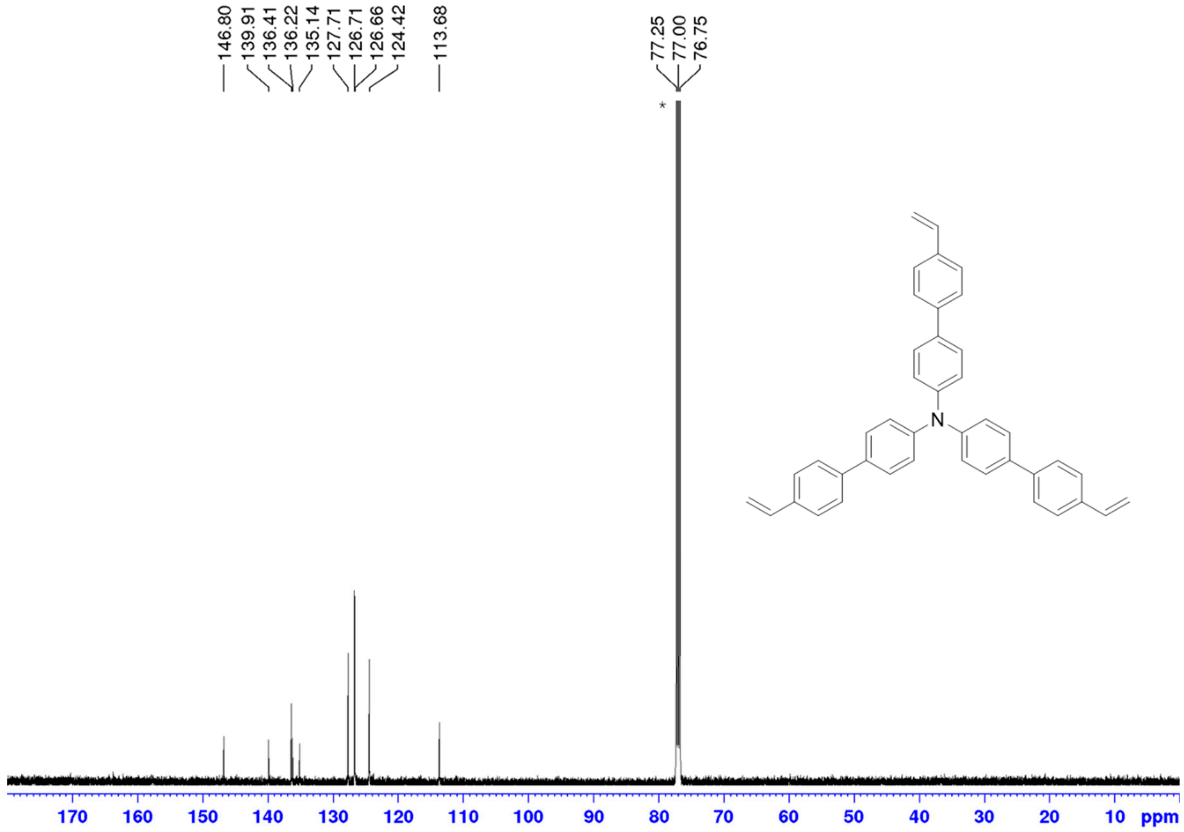

Figure S15. ^{13}C NMR of **M2** in $CDCl_3$

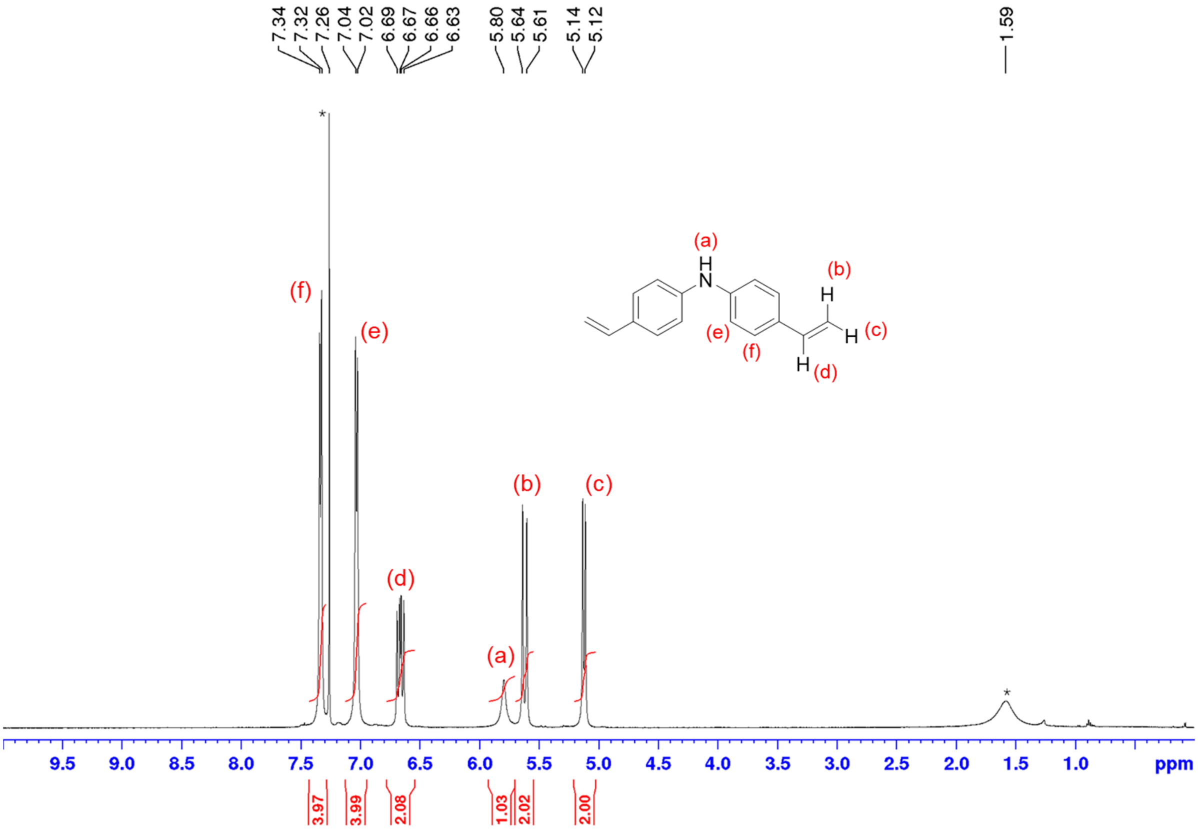

Figure S16. ^{1}H NMR of compound **1** in $CDCl_3$

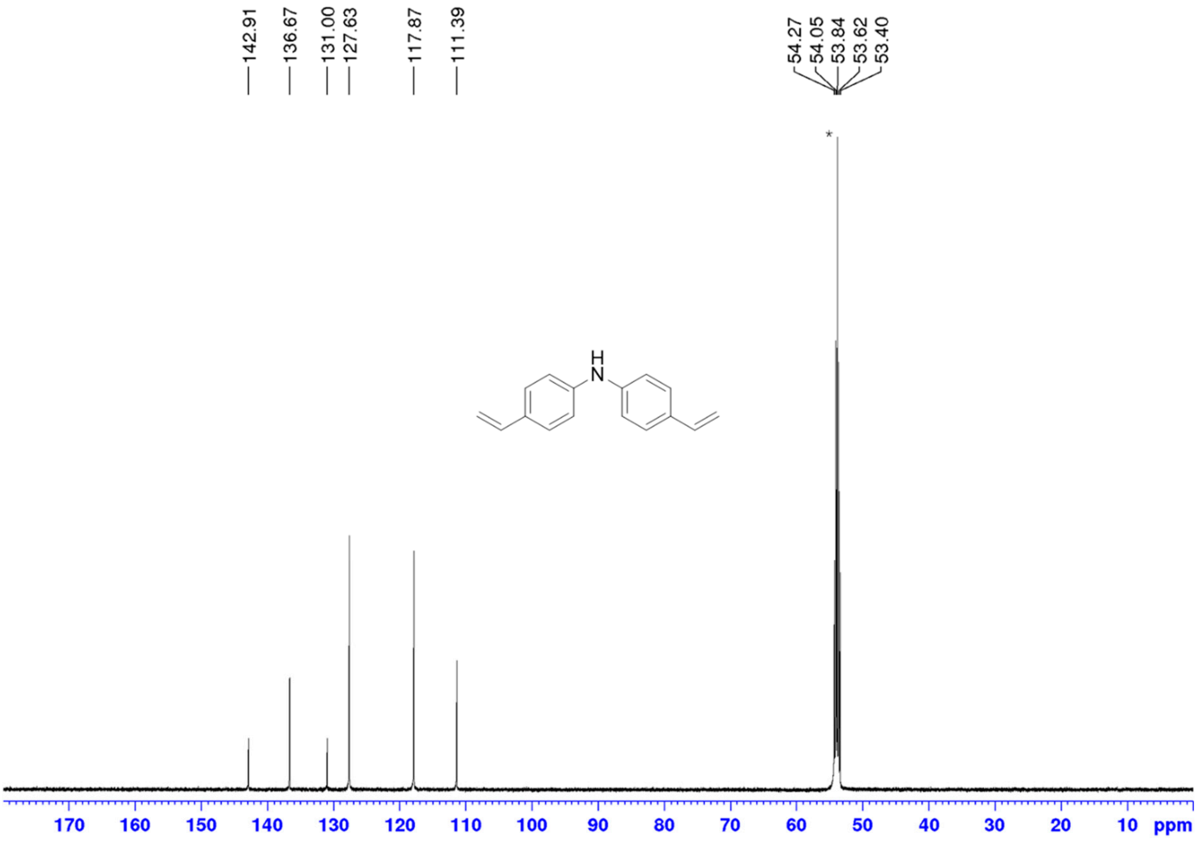

Figure S17. ^{13}C NMR of compound **1** in CD_2Cl_2

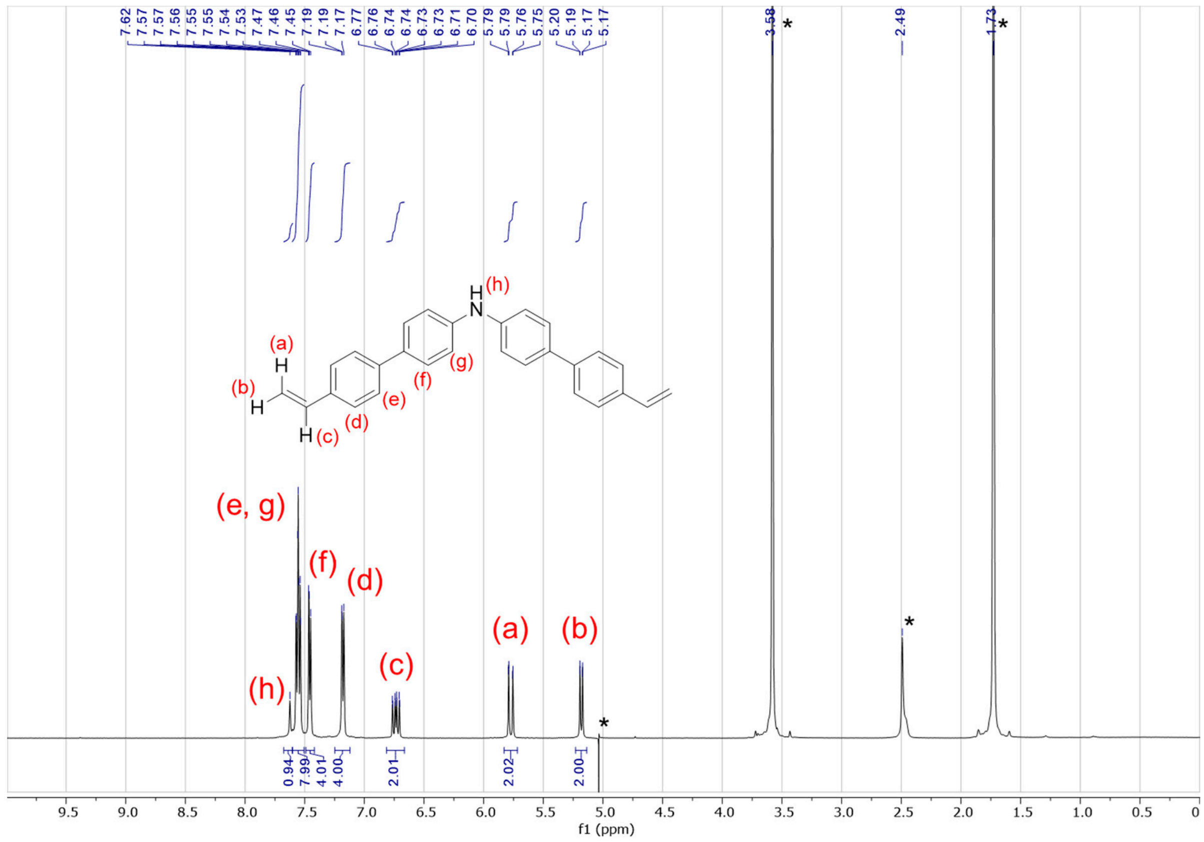

Figure S18. ^{1}H NMR of compound **2** in THF-d8

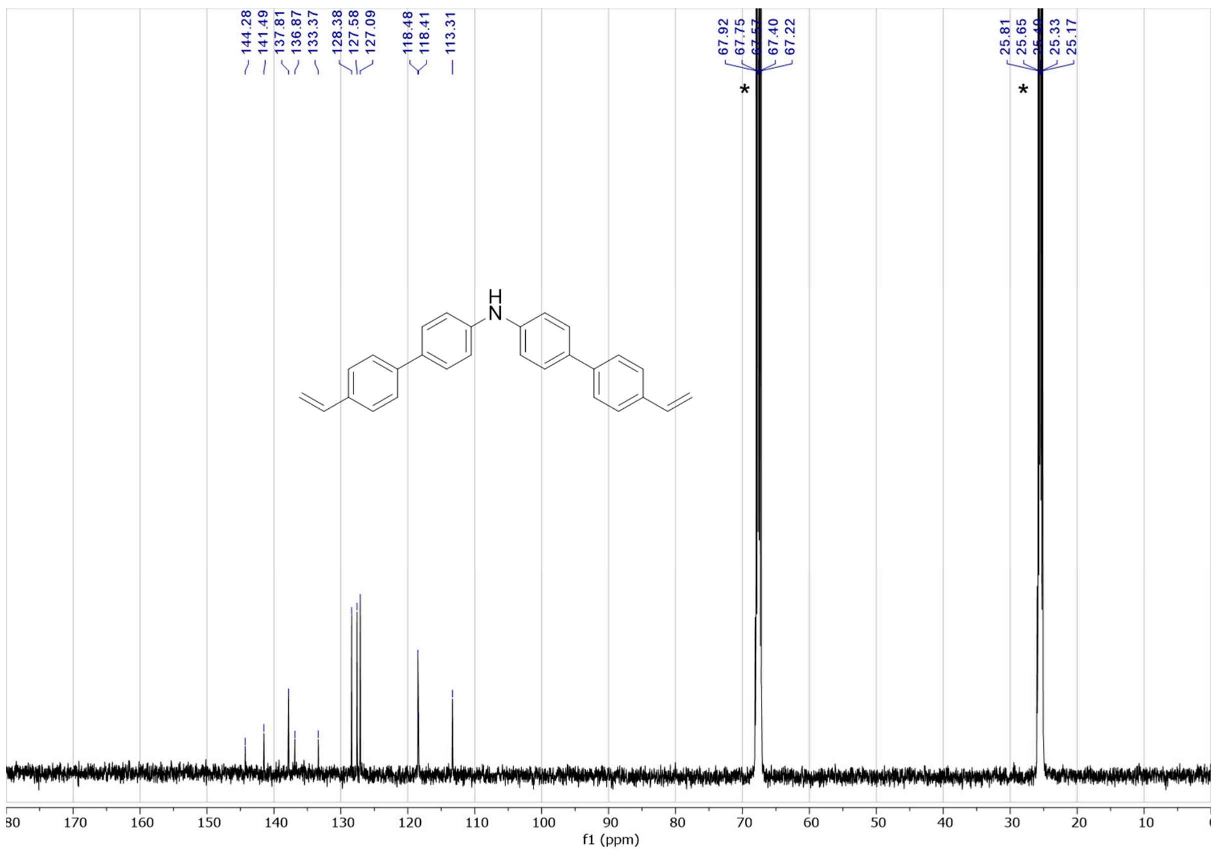

Figure S19. ^{13}C NMR of compound **2** in THF-d8

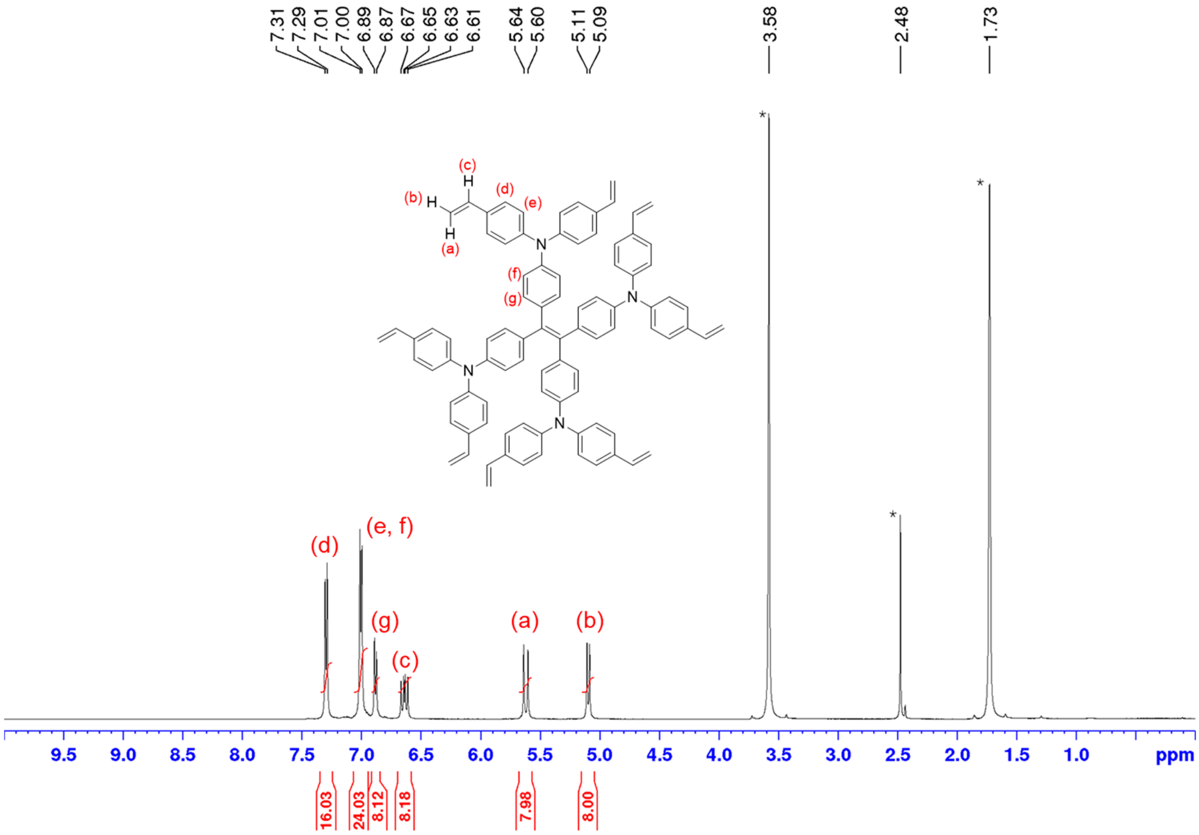

Figure S20. ^{1}H NMR of **M3** in THF-d8

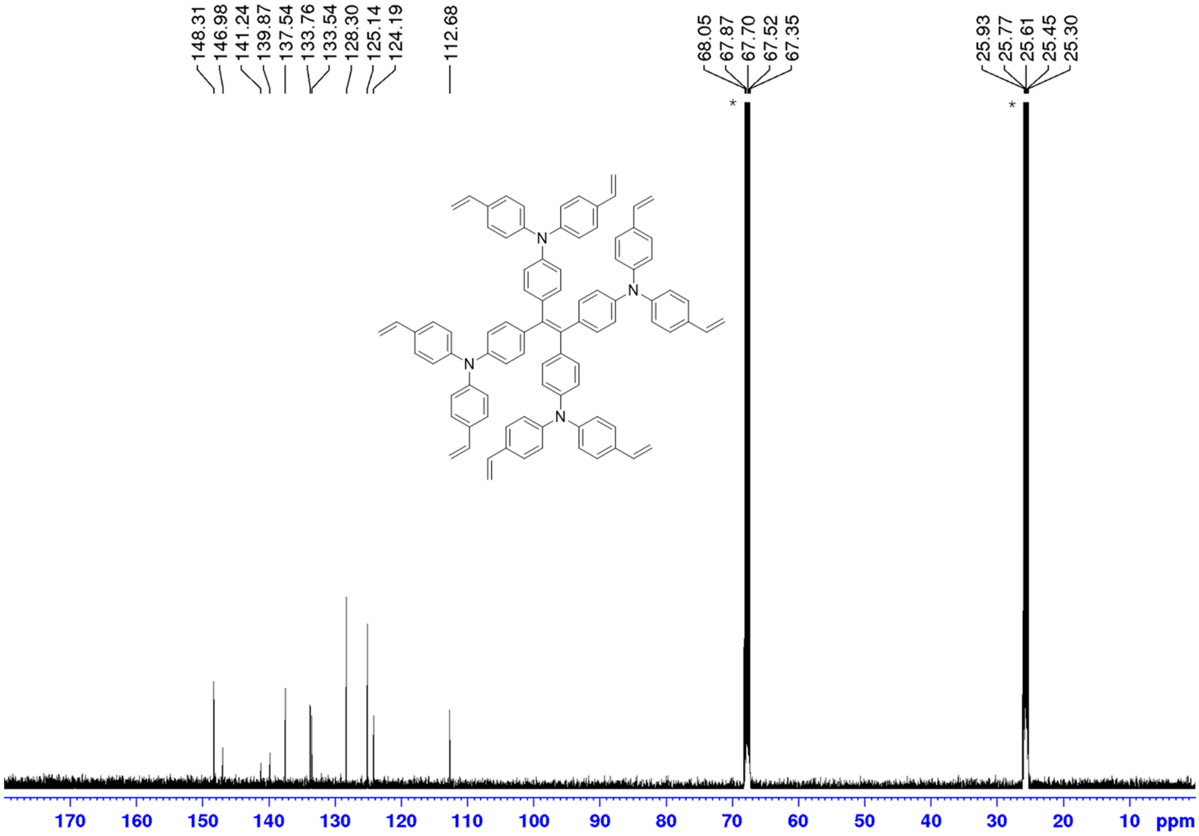

Figure S21. ^{13}C NMR of **M3** in THF-d8

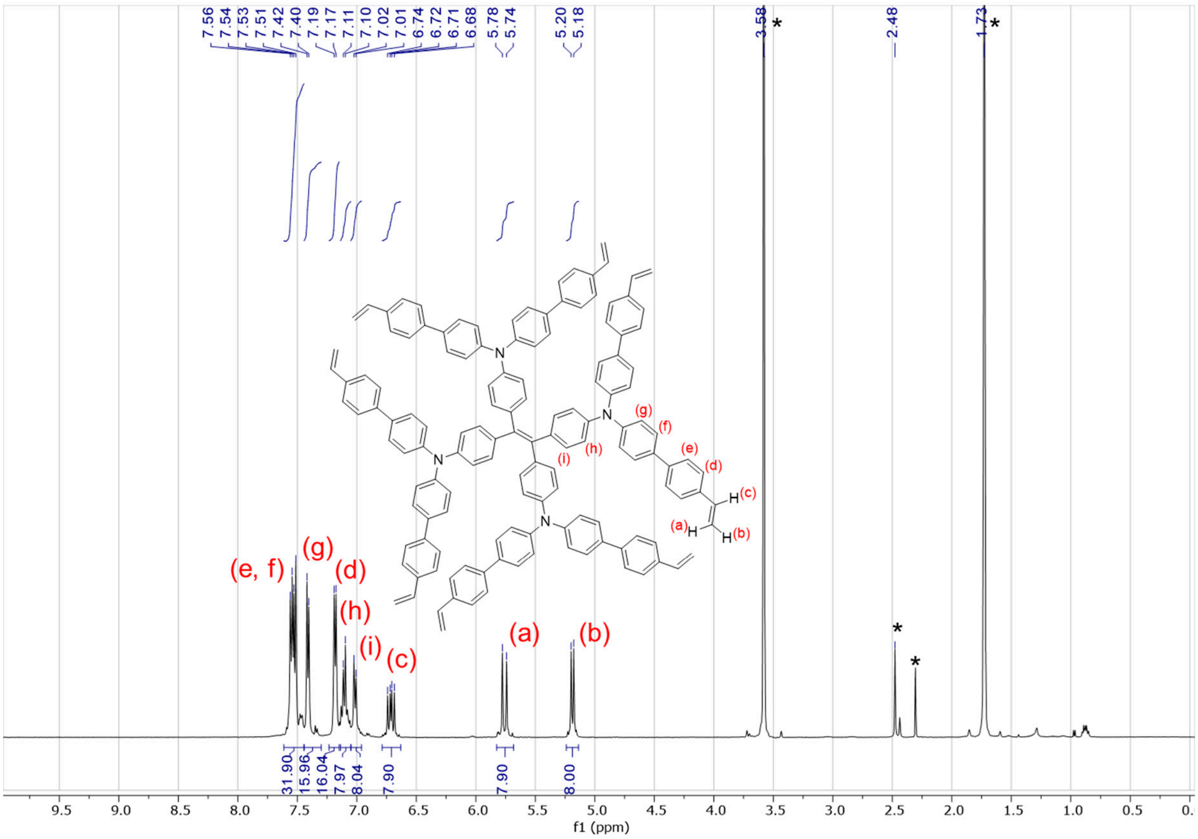

Figure S22. ^{1}H NMR of **M4** in THF-d8

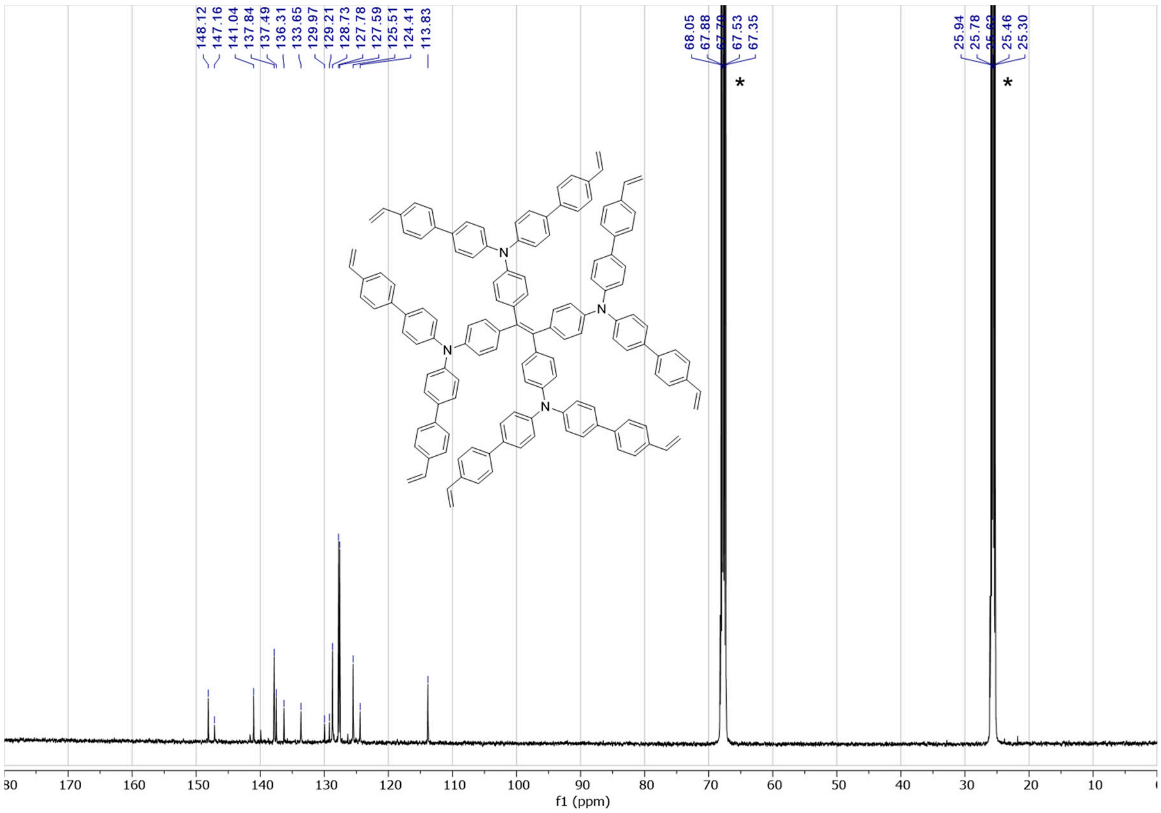

Figure S23. ^{13}C NMR of **M4** in THF-d8

ANNEX 2: High Resolution Mass Spectrometry (HRMS)

Compound Table

Label	Tgt Score	Mass Error (ppm)	Tgt Formula	Obs. RT	Ref. Mass	Obs. Mass
Cpd 1: C24 H21 N; 0.108	96.78	0.33	C24 H21 N	0.108	323.1674	323.16751

Obs. *m/z*	Obs. RT	Obs. Mass	Tgt Formula	Tgt Mass	Tgt Mass Error (ppm)	Find Cpds Algorithm
324.1748	0.108	323.16751	C24 H21 N	323.1674	0.33	Find by Formula

Compound Chromatograms

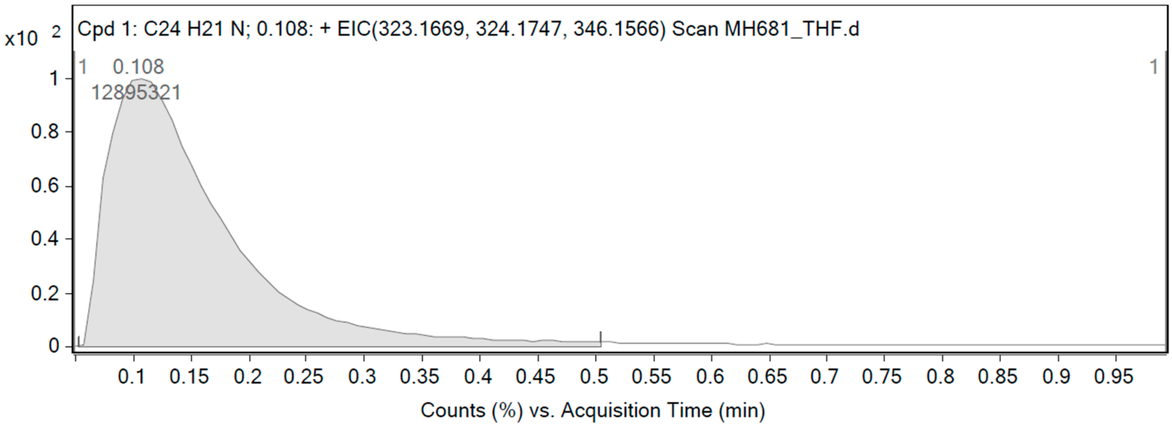

Figure S24. HRMS of **M1**

Compound Table

Label	Tgt Score	Mass Error (ppm)	Tgt Formula	Obs. RT	Ref. Mass	Obs. Mass
Cpd 1: C42 H33 N; 0.888	94.65	0.95	C42 H33 N	0.888	551.2613	551.26182

Obs. *m/z*	Obs. RT	Obs. Mass	Tgt Formula	Tgt Mass	Tgt Mass Error (ppm)	Find Cpds Algorithm
551.2609	0.888	551.26182	C42 H33 N	551.2613	0.95	Find by Formula

Compound Chromatograms

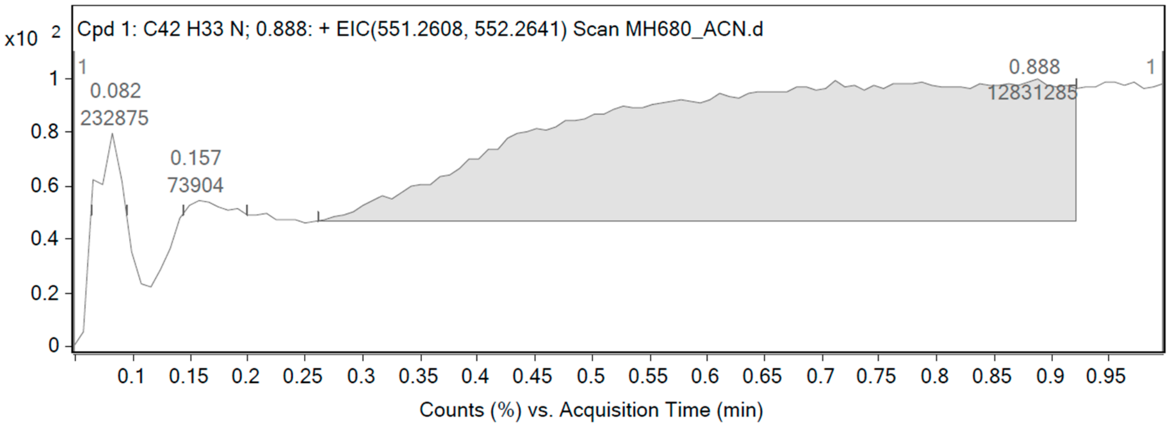

Figure S25. HRMS of **M2**

Compound Table

Label	Tgt Score	Mass Error (ppm)	Tgt Formula	Obs. RT	Ref. Mass	Obs. Mass
Cpd 1: C16 H15 N; 0.095	98.38	-0.4	C16 H15 N	0.095	221.12045	221.12036

Obs. *m/z*	Obs. RT	Obs. Mass	Tgt Formula	Tgt Mass	Tgt Mass Error (ppm)	Find Cpds Algorithm
222.1277	0.095	221.12036	C16 H15 N	221.12045	-0.4	Find by Formula

Compound Chromatograms

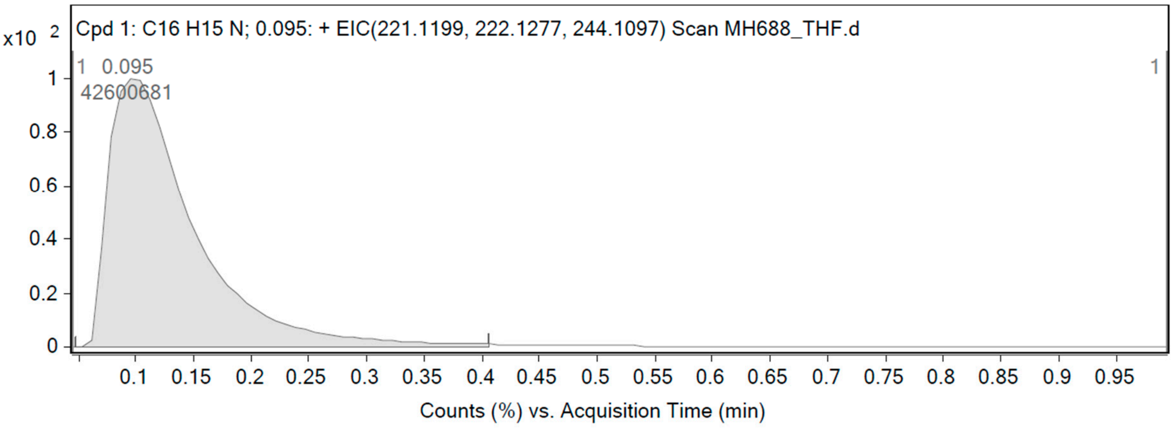

Figure S26. HRMS of compound **1**.

Compound Table

Label	Tgt Score	Mass Error (ppm)	Tgt Formula	Obs. RT	Ref. Mass	Obs. Mass
Cpd 1: C28 H23 N; 0.165	97.94	-1.01	C28 H23 N	0.165	373.18305	373.18267

Obs. *m/z*	Obs. RT	Obs. Mass	Tgt Formula	Tgt Mass	Tgt Mass Error (ppm)	Find Cpds Algorithm
374.19	0.165	373.18267	C28 H23 N	373.18305	-1.01	Find by Formula

Compound Chromatograms

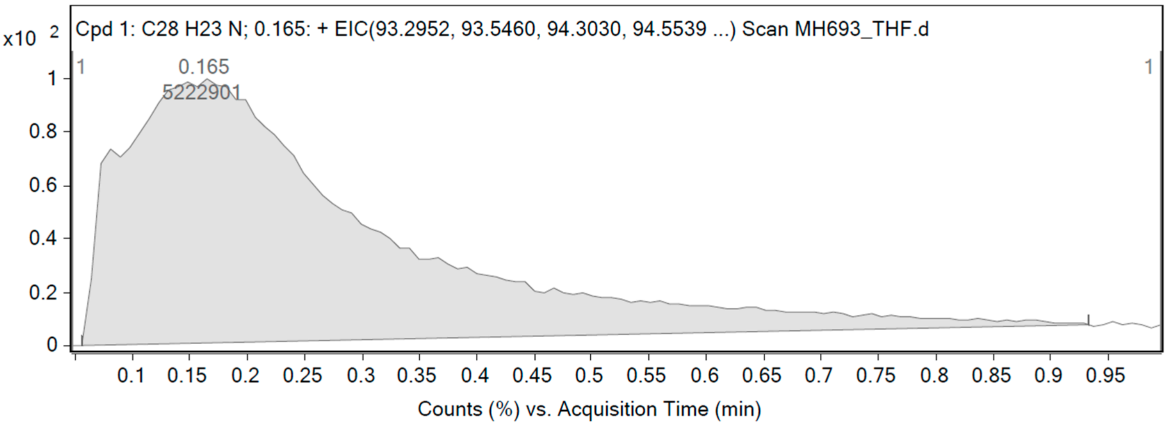

Figure S27. HRMS of compound **2**.

Compound Table

Label	Tgt Score	Mass Error (ppm)	Tgt Formula	Obs. RT	Ref. Mass	Obs. Mass
Cpd 1: C90 H72 N4; 0.077	62.46	-4.35	C90 H72 N4	0.077	1208.5757	1208.57044

Obs. *m/z*	Obs. RT	Obs. Mass	Tgt Formula	Tgt Mass	Tgt Mass Error (ppm)	Find Cpds Algorithm
1209.5779	0.077	1208.57044	C90 H72 N4	1208.5757	-4.35	Find by Formula

Compound Chromatograms

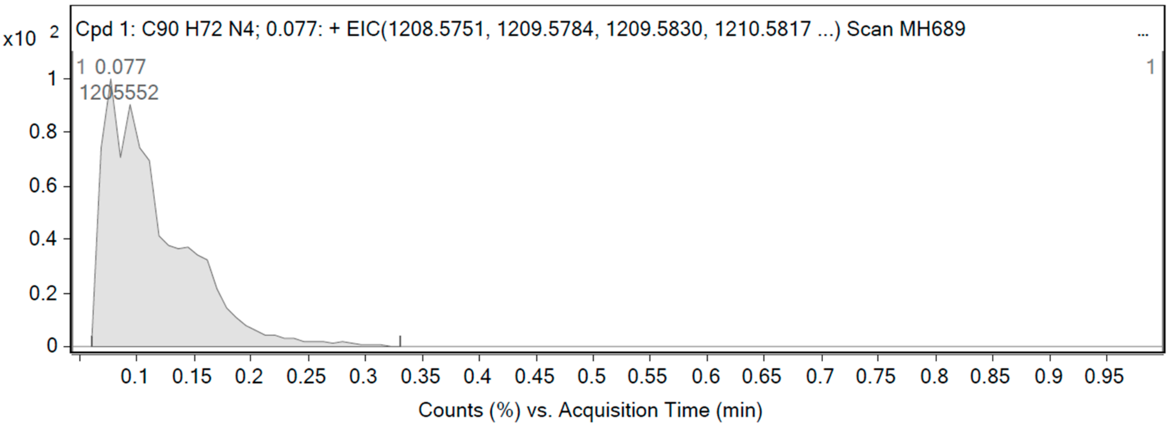

Figure S28. HRMS of **M3**.

Meas. m/z	#	Formula	Calc. Mass	Err [ppm]
1816.8229	1	C138 H104 N4	1816.8256	1.49

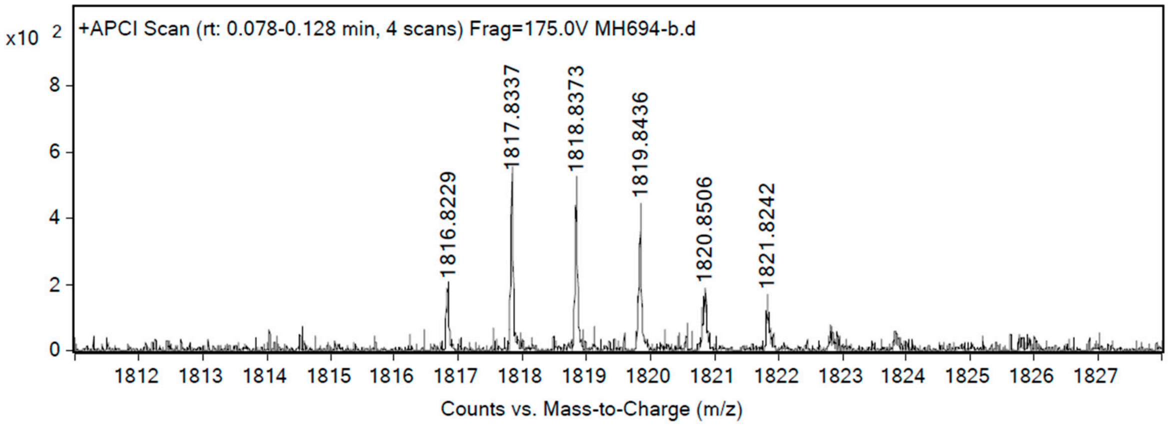

Figure S29. HRMS of **M4**.
